# Erbin protects against sepsis-associated encephalopathy by attenuating microglia pyroptosis via IRE1α/Xbp1s-Ca^2+^ axis

**DOI:** 10.1186/s12974-022-02598-5

**Published:** 2022-09-28

**Authors:** Guoqing Jing, Jing Zuo, Qing Fang, Min Yuan, Yun Xia, Qiyan Jin, Yuping Liu, Yanlin Wang, Zongze Zhang, Wanhong Liu, Xiaojing Wu, Xuemin Song

**Affiliations:** 1 Research Centre of Anesthesiology and Critical Care Medicine, Zhongnan Hospital of Wuhan University, Wuhan, Hubei China; 2 Department of Anesthesiology, Renmin Hospital of Wuhan University, Wuhan, Hubei China; 3 Department of Immunology, School of Basic Medical Sciences, Wuhan University, Wuhan, Hubei China

**Keywords:** Erbin, NLRP3, Pyroptosis, Sepsis-associated encephalopathy, Microglia

## Abstract

**Background:**

Microglia pyroptosis-mediated neuroinflammation is thought to be the crucial pathogenesis of sepsis-associated encephalopathy (SAE). Erbin has been reported to be associated with various inflammatory diseases. However, the role of Erbin in SAE and the relationship between Erbin and microglia pyroptosis are unknown. In this study, we investigated the promising role and underlying molecular mechanism of Erbin in the regulation of microglia pyroptosis.

**Methods:**

WT and *Erbin* knockout mice underwent cecum ligation perforation (CLP) to induce SAE. Primary mouse microglia and BV2 cells were treated with LPS/nigericin in vitro. Behavioral tests were performed to evaluate cognitive function. Nissl staining and transmission electron microscopy were used to assess histological and structural lesions. ELISA and qPCR were carried out to detect neuroinflammation. Western blot and immunofluorescence were used to analyze protein expression. Flow cytometry and confocal microscopy were utilized to observe the Ca^2+^ changes in the cytoplasm and endoplasmic reticulum (ER). To further explore the underlying mechanism, STF083010 was administered to block the IRE1α/Xbp1s pathway.

**Results:**

*Erbin* deletion resulted in more pronounced neuronal damage and cognitive impairment in mice that underwent CLP. *Erbin* knockout promoted microglial pyroptosis and inflammatory cytokines secretion in vivo and in vitro, which was mediated by activation of the IRE1α/Xbp1s. Treatment with the selective inhibitor STF083010 significantly inhibited IRE1α/Xbp1s pathway activity, decreased intracytoplasmic Ca^2+^, attenuated microglial pyroptosis, reduced pro-inflammatory cytokine secretion, lessened neuronal damage, and improved cognitive function.

**Conclusions:**

In SAE, Erbin inhibits IRE1/Xbp1s pathway activity and reduces the ER Ca^2+^ influx to the cytoplasm, reducing microglial pyroptosis.

**Supplementary Information:**

The online version contains supplementary material available at 10.1186/s12974-022-02598-5.

## Introduction

Sepsis-associated encephalopathy (SAE), a common and severe complication in patients with sepsis, is a diffuse brain dysfunction caused by sepsis, mainly manifests as long-term cognitive impairments and psychiatric diseases, and is closely associated with increased morbidity and mortality [1, 2]. Cognitive deficits are found in about 70% of survivors who recovered from severe systemic infections [3]. Although the pathogenesis of SAE is not fully elucidated, sepsis-induced neuroinflammation is thought to be an essential contributor to cognitive dysfunction [4].

Microglia are the central innate immune cells and the primary producers of pro-inflammatory cytokines in the brain. Therefore, they are the focus of research when investigating neuroinflammation. Microglia are rapidly activated in response to various stimuli, including infectious, pathological stimuli, or Aβ peptides. Activated microglia can secrete substantial pro-inflammatory cytokines, such as TNF-α, IL-6 and IL-1β. This amplified neuroinflammation in the brain exacerbates neuronal damage or even death, resulting in behavioral and psychological symptoms of SAE [5].

Activation of the NLRP3 inflammasome and the occurrence of pyroptosis in microglia are implicated in the pathogenesis of SAE [6, 7]. Microglia are the main cells where pyroptosis occurs in the central nervous system (CNS) [8]. NLRP3 inflammasome, the most well-characterized inflammasome, is a multi-protein complex composed of Nod-like receptors (NLRs), apoptosis-associated speck-like proteins containing a caspase recruitment domain (ASC), and pro-Caspase-1 [9]. NLRs recognize multiple stimuli to form complexes to cleave pro-Caspase-1 into activated Caspase-1. Activated Caspase-1 then cleaves the pore-forming protein gasdermin D (GSDMD), pro-IL-1β, and pro-IL-18, leading to pyroptosis and IL-1β, IL-18 secretion. Pyroptosis is a pro-inflammatory programmed cell death mediated by GSDMD-N (N-terminal fragment of GSDMD) cleaved by Caspase-1. GSDMD-N binds to membranes to form membrane pores and promotes the release of inflammatory factors, notably IL-1β [10, 11]. Pyroptosis is closely related to neuroinflammatory diseases, such as subarachnoid hemorrhage (SAH), cerebral venous sinus thrombosis (CVST), and spinal cord injury (SCI). TREM-1 exacerbates neuroinflammatory injury via microglia-mediated pyroptosis in SAH [12]. NLRP3 inflammasome activation and pyroptosis in microglial modulated by the cGAS–STING pathway are involved in the pathogenesis of CVST [13]. CD73 alleviates GSDMD‐mediated microglia pyroptosis in SCI through PI3K/AKT/Foxo1 signaling [14]. However, the mechanisms by which it occurs are not well-understood in SAE.

Recent evidence has suggested that endoplasmic reticulum (ER) stress is involved in NLRP3 inflammasome activation [15]. ER is responsible for protein synthesis and processing and cellular Ca^2+^ homeostasis. Several stimuli, such as ischemia, hypoxia, and bacterial infections, can induce the accumulation of misfolded proteins and result in ER stress [16]. Then ER initiates three signaling pathways mediated by ER-resident protein folding sensors inositol-requiring enzyme 1 alpha (IRE1α), PKR-like endoplasmic reticulum kinase (PERK), and activating transcription factor 6 (ATF6) to restore ER homeostasis [17]. Hyperactivation of ER stress leads to Ca^2+^ release and oxidative stress, which triggers a downstream cascade leading to inflammation [18]. IRE1α is the most conserved ER stress sensor and has both kinase and RNase activity. IRE1α autophosphorylates, activating RNase activity, and splices X-box-binding protein 1 (XBP1) to form spliced XBP1 (XBP1s) under ER stress. Plentiful studies have proved that IRE1α signaling could activate NLRP3 inflammasome and pyroptosis in multiple diseases such as mellitus [19], hypoxic-ischemic brain injury [20], and nonalcoholic fatty liver disease [21]. Nevertheless, whether it is involved in the pathogenesis of SAE remains unknown.

Erbin, first discovered as an interacting protein of ErbB2, is a member of the epidermal growth factor receptor family. Recently, the role of Erbin in various diseases and signaling pathways has been extensively studied. In most cases, Erbin is involved in regulating multiple cell signaling as a negative regulator, including MAP kinase pathway [22], NF-κB [23], Ras–Raf–ERK signaling pathway [24], and transforming growth factor-beta-signaling pathways [25]. Erbin deficiency could negatively regulate NOD2-mediated NF-κB activation and overexpressed Erbin in mouse embryonic fibroblasts, significantly inhibiting the production of pro-inflammatory cytokines induced by MDP [26]. Shen et al. [27] found that in the DSS-induced mouse colitis model, the expression of Erbin in colon tissue was reduced considerably, and Erbin-deficient mice were more prone to small intestinal inflammation. The above studies show that Erbin plays an essential role in inflammatory diseases. However, whether Erbin regulates the microglia-mediated neuroinflammation and its role in SAE has not yet been elucidated.

We hypothesized that Erbin could attenuate NLRP3 inflammasome activation and inhibit pyroptosis in microglia, reducing neuroinflammation in SAE. In the present study, we first validated the role of Erbin in SAE by behavioral tests. Then, we found that Erbin suppressed microglia pyroptosis by restricting the flow of Ca^2+^ from ER to the cytoplasm. Finally, we confirmed that Erbin inhibits neuroinflammation and improves cognitive function by negatively regulating IRE1α/Xbp1s pathways.

## Materials and methods

### Animals

Male C57BL/6 J aged 6–8 weeks (weighing 20–25 g) were enrolled in this study. WT mice were obtained from Hubei Province Center for Animal Experiments, and *Erbin*-knockout (*Erbin*^*−/−*^) mice were purchased from Wuhan Xianran Biotechnology Co., Ltd. (Contract Number: Mouse-2018-9-25-WJB-3, China). Mice were housed at 22 °C with a relative humidity of 50–60% and a 12 h of light/dark cycle; food and water were available ad libitum. All animal care and experiments were performed according to the ethical regulations set by the Animal Experimentation Committee of Wuhan University (WQ20210298).

### Sepsis model and drug treatment

Laparotomy was performed to isolate the cecum after intraperitoneally anesthetized with pentobarbital sodium (50 mg/kg). The cecum was ligated using 3.0 silk and then punctured twice with a 21 G needle. Next, the abdominal incision was sutured, and the animals received 1 ml of 0.9% normal saline as resuscitation subcutaneously. Mice in the Sham group underwent the same operation without the ligation and puncture of the cecum. For further studies on the effect of STF083010 on septic mice, intranasal administration was performed according to the previously used protocol [28]. 1 h after cecal ligation and puncture (CLP), the mice were anesthetized with a 2% isoflurane and positioned supine. A total volume of 5 μl of STF083010 (2.5 mg/kg, MedChemExpress) was instilled into the nasal cavity of mice. Repeated dosing once daily for three consecutive days. The Sham group was given the same volume of DMSO at the same time.

### Behavioral tests

#### Open field test (OFT)

In this study, an OFT was performed to evaluate the locomotor activity of mice. Each mouse was gently placed in the center of the box (40 × 40 cm) and allowed to explore the apparatus for 5 min. The movement of mice was recorded, and the total traveled distance was analyzed.

#### Novel object recognition test (NORT)

The NORT was carried out to evaluate recognition memory based on a previous study reported [29]. Before the experiment, each mouse was gently placed in a field arena (30 × 30 cm) for 3 min to acclimate to the environment. Then, the mice were randomly placed in the center of the arena to explore two identical objects for 5 min each. 24 h post the training, one of the familiar objects was replaced with a novel object, and each mouse was allowed to explore for 5 min. The preference index was calculated as [time spent exploring the novel object/time spent exploring the two objects] × 100%.

### Morris Water Maze (MWM) test

The MWM test was implemented 10 days after the operation to assess the spatial learning and memory of the mice. Briefly, the mice were trained for four consecutive days, followed by a probe trial on the 5th day. The MWM consisted of a round steel pool (diameter of 1.2 m, height of 0.6 m) filled with water maintained at 23°C and a hidden platform (diameter of 0.1 m) located in the southwest quadrant of the pool approximately 1 cm below the water surface. Titanium dioxide was used to make the water opaque. Each mouse was randomly placed into each pool quadrant every day during the training period. Mice were allowed to find the platform for 60 s, and the latency to the platform was recorded in each trial. If the mouse failed to find the platform within 60 s, it was guided to the platform to rest for 10 s. On the 5th day, removed the platform from the swimming pool and released the mice into the water from the northeast quadrant. Each mouse was allowed to swim freely for 60 s, and the number of platform crossings and the seconds of search time in the target quadrant were recorded.

### Nissl staining

Nissl staining was performed to evaluate neuronal damage and loss. After paraffin embedding and sectioning (4 μm), brain tissues were stained with a 1% toluidine blue solution (Boster Biotechnology, China).

### Immunofluorescent staining

For primary microglia, after indicated treatment, cells were fixed with 4% paraformaldehyde for 30 min and then permeabilized by 0.5% Triton X-100 for 30 min. Following blocking with 5% BSA for 1 h, cells were incubated with indicated primary antibodies overnight at 4°C. After three washes with PBS, cells were stained with DayLight 488/CY3-conjugated secondary antibodies (1:400, Abbkine) for 1 h. DAPI was used to stain nuclei. Images were recorded by Leica confocal microscopy. For brain tissue, double immunofluorescence staining was performed on formalin-fixed, paraffin-embedded brain sections. After blocking with 5% BSA, 4 μm thick paraffin brain slices were incubated with rabbit anti-Caspase-1 (1:200, Abclonal), rabbit anti-GSDMD (1:200, Abclonal), or mouse anti-Iba-1 (1:200, Abcam) antibodies overnight at 4°C. Then, the slices were incubated with CY3-conjugated duck anti-rabbit or DayLight 488-conjugated duck anti-mouse secondary antibodies (1:400, Abbkine) for 1 h at 37 °C. After washing with PBS, DAPI was used to label the nuclei. The number of double-positive cells was detected under a fluorescence microscope.

### Cell culture and treatment

Primary mouse microglia were obtained from newborn WT and *Erbin*^*−/−*^ mice at postnatal days 1–2 as previously reported [30]. Briefly, the pups were decapitated, removed the meninges, and cut cortices into small pieces. After trypsin enzymatic digestion, cells were filtered by passing through a cell strainer (70 μM pores, Corning, USA), then resuspended in complete DMEM-F12 (GIBCO, USA) with 10% fetal bovine serum (FBS) (GIBCO, USA) and 1%penicillin/streptomycin (Biosharp, China) and seeded on poly-d-lysine-coated T75 flasks. Then, the cells were cultured in an incubator (37 °C, 5% CO_2)_, and the medium was changed every 3–4 days. About 2 weeks later, cells were shaken using a horizontal orbital shaker at 200 rpm for 2 h at a constant temperature (37˚C) to isolate the matured microglia.

The mouse microglia BV2 cell line and mouse hippocampal neuron HT22 cell line were purchased from the Procell Life Science&Technology Co., Ltd (Wuhan, China) and cultured in DMEM (GIBCO, USA) supplemented with 10% FBS and 1% penicillin/streptomycin at 37˚C in a humid air atmosphere containing 5% CO_2_. LPS (E. coli 0111:B4, L2630, Sigma) and nigericin (HY-100381, MedChemExpress) were used to establish the activation of the NLRP3 inflammasome cell model. For NLRP3 inflammasome activation treatment, primary microglia and BV2 cells were treated with LPS (500 ng/ml) for 4 h and then treated with nigericin (10 µΜ) for 45 min. For intervention experiments, primary microglia and BV2 cells were pretreated with 30 µΜ STF083010 (dissolved in DMSO) (HY-15845, MedChemExpress) for 2 h, followed by treatment with LPS and nigericin. DMSO was designed as vehicle control for treatment conditions.

### Establishment of microglia-conditioned medium (CM)

Primary microglia were plated onto 6-well plates and then treated with DMSO, STF083010, or LPS/nigericin at the appointed times. 45 min after nigericin was added, replaced the old medium with fresh medium, and continued to culture the microglia for 24 h. Then, the supernatant was collected and centrifuged at 1000 rpm for 5 min. Next, the HT22 cells’ medium was replaced with the collected CM and cultured for 12 h.

### Plasmids and siRNA transfection

Small interfering RNA against Erbin (*siErbin*) was purchased from Tsingke Biotechnology Co., Ltd (Nanning, China) and transfected into BV2 cells using jetPRIME (Polyplus transfection reagent, France) according to the manufacturer's instructions. After 48 h, the cells were used for further experiments. The following sequences were used: *siErbin* sense: 5'-GCAAGCGGUGUCCUUGUUATT-3', negative control siRNA (*siNC*) sense: 5'-UUCUCCGAACGUGUCACGUTT-3'.

The ER-targeted plasmid CMV-ER-LAR-GECO1 (#61,244, Addgene), a sensitive single-wavelength Ca^2+^ indicator for detecting Ca^2+^ dynamics within the ER of mammalian cells [31], was a gift from Han song. Moreover, the changes in Ca^2+^ concentration in ER were observed by a confocal microscope or a flow cytometer.

### Cell viability

Primary microglia were seeded into a 96-well plate (1 × 10^4^ cells/well), and LPS was added to the cells for 4 h and then stimulated with 10 μM nigericin for 45 min. HT22 cells were plated in 96-well plates at a density of 10^3^ cells per well, cultured for 24 h, then replaced the media with microglia-CM, and cultured for another 12 h. Finally, 10 µL CCK-8 reagents (Biosharp, China) were added per well. The absorbance at 450 nm was measured to calculate the cell viability using a microplate reader.

### LDH assay

LDH is an indicator of cell membrane integrity and is also used to indirectly indicate the onset of pyroptosis [32]. The culture medium was collected and centrifuged at 500×*g*, 4 °C for 10 min. Then, the supernatant was transferred into 1.5 mL EP tubes. LDH release was measured with an LDH assay kit (Beyotime, China) according to the manufacturer’s manual.

### Reverse transcription real-time quantitative polymerase chain reaction (RT q-PCR)

Total RNA was extracted from BV2 cells with TRIZOL Reagent (Invitrogen, USA). RNA was reverse-transcribed to complementary DNA using the RT-qPCR Fast Master Mix (Vazyme, China). Real-time fluorescence quantitative PCR detection was performed according to the manufacturer's instructions. The GAPDH gene was used as an internal control for IL-1β, TNF-α, and IL-6 mRNA expressions analysis. The gene expression was analyzed using the 2 − ΔΔCt method for quantification. All gene primer sequences were used as follows: IL-1β, forward 5′-GAAATGCCACCTTTTGACAGTG-3′, and reverse 5′-TGGATGCTCTCATCAGGA.

CAG-3′; TNF-α, forward 5′-GGCATGGATCTCA AAGACAAC-3′ and reverse 5′-TGGATGCTCTCATCAGGACAG-3′; IL-6, forward 5′-CATGTTCTCTGGGAAAT.

CGTGG-3′ and reverse 5′-GTACTCCAGGTAGCTATGGTAC-3′; GAPDH, 5′-AGGT.

CGGTGTGAACGGATTTG-3′ and reverse 5′-TGTAGACCATGTAGTTGAG GTA-3′.

### Flow cytometry

Fluo4-AM (1 μM, Beyotime, China) was used to indicate the cytoplasmic Ca^2+^ concentration. Cells were incubated with Fluo4-AM solution for 30 min in a cell incubator and washed three times using PBS. CMV-ER-LAR-GECO1 was used to indicate ER Ca^2+^. 48 h after transfection of plasmids. Primary microglia were treated with DMSO, STF083010, or LPS/nigericin according to the appointed time. Then discarded medium and washed three times with PBS. The green (intracytoplasmic Ca^2+^, FITC) and red (ER Ca^2+^, PE) fluorescence were measured by flow cytometry.

### Transmission electron microscopy (TEM)

After transcardial perfusion with 50 mL of saline and followed by 50 mL of 4% paraformaldehyde, about 1 mm^3^ hippocampal CA1 tissues were collected and fixed in 2.5% glutaraldehyde, dehydrated through a grade ethanol series and propylene oxide, and then embedded in Epon. The ultrathin sections (70 nm) were placed onto 200 mesh copper grids and then stained with 4% uranyl acetate and 0.04% lead citrate. The stained sections were observed under a transmission electron microscope (Hitachi H-600, Hitachi, Tokyo, Japan). The thickness of the postsynaptic density (PSD) was assessed as the length of the vertical line from the postsynaptic membrane to the most convex part of the synaptic complex. The width of the synaptic cleft was evaluated by measuring the narrowest and widest parts of the synapse and then taking the average of these values.

### Western blot analysis

The total protein content was extracted from hippocampal tissue or cultured cells using RIPA lysis buffer (Beyotime, China) containing PMSF (BioSharp, China) and phosphatase inhibitor (Beyotime, China). The protein concentration in the supernatant was quantified by a BCA protein assay (Beyotime, China). Equal amounts of protein samples were then separated by SDS–PAGE and transferred to PVDF (MilliPole, UK). PVDF membranes were then blocked with 5% BSA for 2 h at room temperature and incubated with primary antibodies (Erbin (NBP2-56,104, Novus), NLRP3 (A5652, Abclonal), GSDMD (A10164, Abclonal), Caspase-1 (AG-20B-0042, adipogenic), Iba-1 (ab178846, Abcam), IRE1α (3294, CST), p-IRE1α (ab48187, Abcam), Xbp1s (24,868–1-AP, proteintech), PSD95 (3409, CST), Synaptophysin (AF0257, affinity), Synapsin-1 (5297, CST), β-actin (HRP-60008, proteintech)) antibody overnight at 4 °C. After incubation, membranes were washed with TBST and then incubated with the corresponding HRP-conjugated secondary antibodies for 2 h. Then, washed the membrane with TBST. Finally, immunoreactive bands were detected with an enhanced chemiluminescence detection reagent. Band intensities were quantified by spot densitometric analysis using ImageJ software, and results were normalized to β-actin levels and reported as relative intensities to controls.

### ELISA

The levels of IL-1β, IL-18, TNF-α, and IL-6 in the hippocampus and medium of primary microglia were determined using an ELISA kit (Beijing 4A Biotech Co., Ltd) according to the manufacturer's instructions. The absorbance of the samples at 450 nm wavelength was measured with a BioTek microplate reader.

### Statistical analysis

GraphPad Prism 8.3.0 was used to analyze data and construct the graphs. All data were expressed as mean ± SEM. MWM training experiments were analyzed using two-way ANOVA for repeated measures followed by Bonferroni correction for multiple testing. Multiple groups were analyzed by two-way ANOVA followed by the Bonferroni post-tests. *P* < *0.05* was considered statistically significant.

## Result

### Erbin improves survival rate and alleviates cognitive impairment in CLP-induced septic mice

**Fig. 1:**
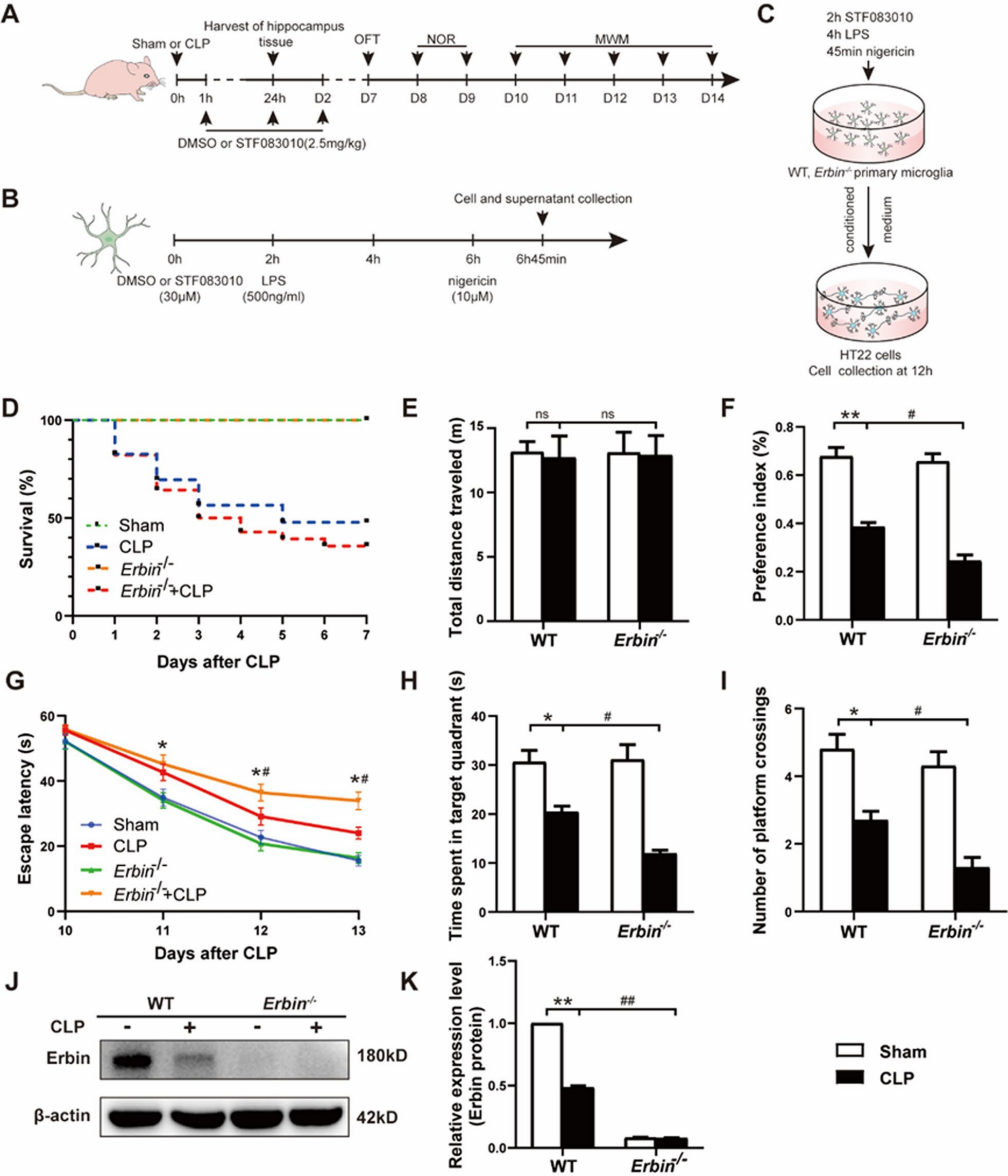
Erbin improves survival rate and alleviates cognitive impairment in CLP-induced septic mice. **A** Experimental design. 6–8 week male C57BL/6 J mice were subjected to sham or CLP operation, then STF083010 (2.5 mg/kg) or DMSO was given at 1 h, 24 h, and 48 h after CLP. The hippocampus of different groups was collected for tests 24 h. The OFT, NORT, and MWM tests were carried out from the 7th to the 14th day. **B** Primary microglia and BV2 cells were incubated with STF083010 (30 µΜ) or DMSO, then were added LPS (500 ng/ml) 2 h later, and finally were administrated with nigericin (10 µΜ) 4 h after adding LPS. The cells and culture medium supernatants were collected for testing 45 min after incubation.** C** Primary microglia were plated onto 6-well plates and then treated with DMSO, STF083010, or LPS/nigericin at the appointed times. 45 min after nigericin was added, new media were adopted to replace the old medium, and the microglia were continued to culture for 24 h. Then the supernatant was collected and added into the HT22 cells’ medium and cultured for 12 h. **D** 7-Day survival rate of mice after CLP. **E** Traveled distance of the four groups of mice treated in the OFT. **F** Preference index of the four groups of mice in the NORT. **G** Escape latency, **H** time spent in the target quadrant, and **I** numbers of crossings through the platform were recorded in each group (n = 10 per group). **J**, **K** Erbin expressions in the hippocampus after CLP were analyzed by western blot (n = 3 per group). *p < 0.05, **p < 0.01 vs. Sham group; ^#^*p* < 0.05, ^##^*p* < 0.01 vs. CLP group

To investigate the physiological role of Erbin in SAE, we established a CLP sepsis model by using WT and *Erbin*^−/−^ mice. Mice subjected to CLP operation were lethargic, with reduced activity, slow movement, erect back hair, thick secretions on the eyelids, fecal adhesions at the anus of the mice, and cloudy urine. The dissection of the dead mice showed that the abdominal cavity was filled with turbid exudate and the intestinal tract was adherent. After 5 days, the state of the surviving mice began to improve. Mice in the sham-operated group returned to normal after waking up after surgery. First, we recorded the survival rate of mice throughout 7 days after CLP, and all WT and *Erbin*^−/−^ sham-operated mice were normal with 100% survival. Survival analysis showed that compared with the Sham group, the survival rate of the CLP group was significantly decreased, and the 7-day survival rate was about 47.8% (11 of 23 mice survived), with most deaths occurring in the first 3 days. In addition, *Erbin* depletion further exacerbated acute mortality in septic mice, and the survival rate was only 35.7% (10 of 28 mice survived) (Fig. 1D).

To further study the effect of Erbin on cognitive function, behavioral tests were carried out 7 days after CLP. There was no significant difference in the total distance traveled among the four groups in the OFT (Fig. 1E), suggesting that sepsis does not affect general motor activity. The NORT was carried out to evaluate recognition memory. As revealed in Fig. 1F, compared with the Sham group, the preference index of mice in the CLP group decreased significantly, and the index further dropped when *Erbin* was knocked out. We next performed the MWM test to assess mice's spatial memory and learning ability. Latencies did not differ between the four groups on the first day of MWM training, and latencies in all groups gradually decreased over the next 3 days. However, the 3–4 day training results showed that the latencies of both CLP groups were longer than their respective sham controls, and *Erbin*^*−/−*^ septic mice had longer latencies than mice in the CLP group. Compared with the Sham group or *Erbin*^*−/−*^ group (Fig. 1G), mice in both CLP groups stayed in the target quadrant for less time (Fig. 1H) and crossed the platform fewer times (Fig. 1I). In addition, *Erbin*^*−/−*^ septic mice spent less time in the target quadrant and displayed fewer platform crossings than mice in the CLP group. These results demonstrate that SAE occurred after 7 days of CLP, and *Erbin* knockout aggravates cognitive dysfunction in septic mice.

Recent studies have shown that Erbin is a crucial regulator of inflammatory diseases. So we study the expression pattern of Erbin in the hippocampus. Results by western blot showed that Erbin was down-regulated during sepsis induced by CLP (Fig. 1J–K), suggesting that down-regulation of Erbin may be associated with SAE in mice.

### Erbin alleviates SAE by attenuating neuronal damage, improving the synaptic structure, and increasing synaptic protein expression

**Fig. 2:**
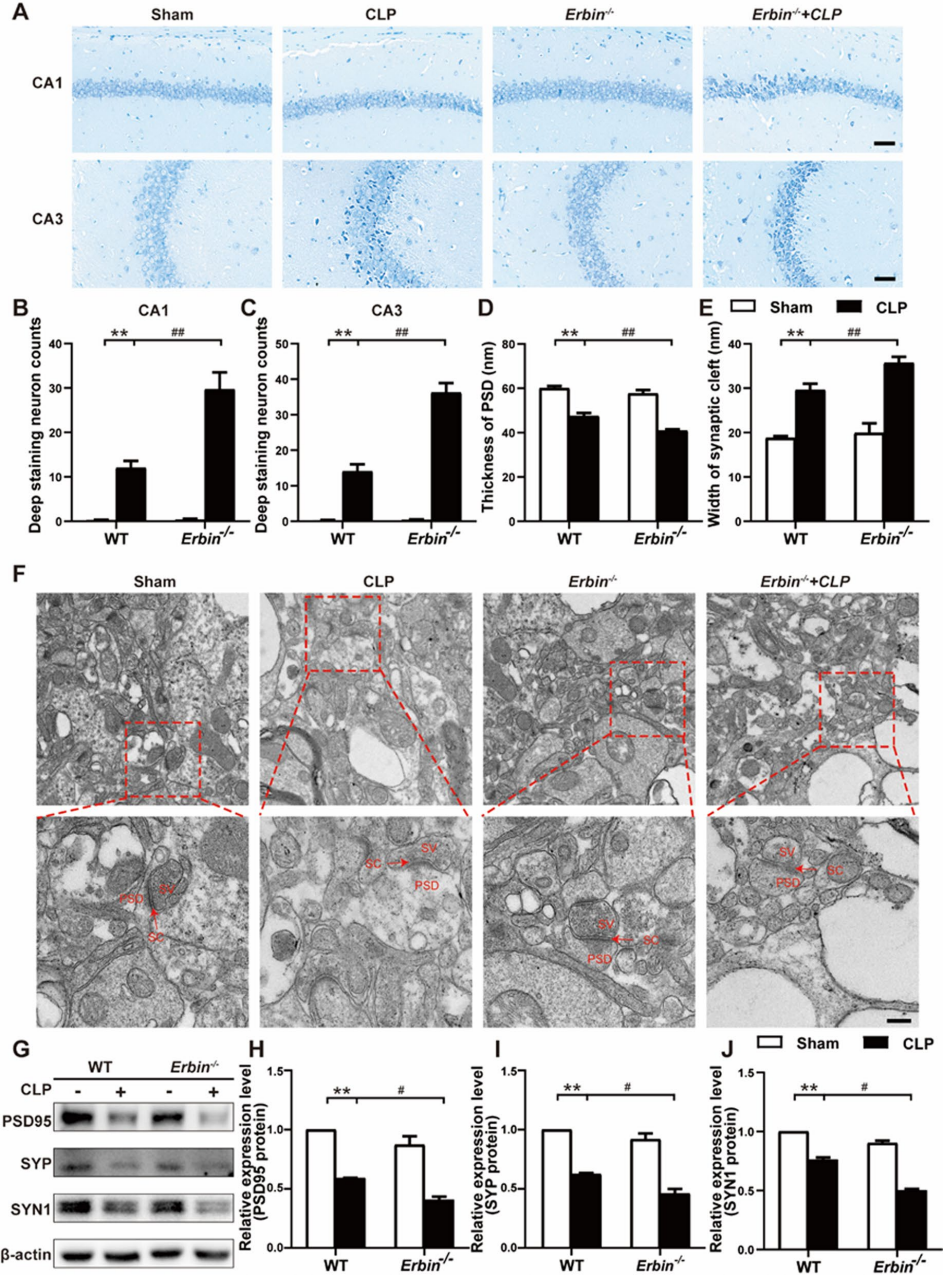
Erbin alleviates SAE by attenuating neuronal damage, improving the synaptic structure, and increasing synaptic protein expression. **A**–**C** Neuronal damage of the hippocampal region was assessed by Nissl staining. Scale bar, 50 μm. **D**, **E** Image analysis of the thickness of PSD and the width of the synaptic cleft. **F** Synaptic structural changes were observed in TEM. Scale bar, 50 nm. SV, Synaptic vesicles; SC, Synaptic cleft; PSD, Postsynaptic density. **G**–**J** Levels of PSD95, SYP, and SYN1 in hippocampal tissue were measured by western blot. Data are representative of three independent experiments. *p < 0.05, **p < 0.01 vs. Sham group; ^#^*p* < 0.05, ^##^*p* < 0.01 vs. CLP group

Neuronal injury, especially in the hippocampus, alterations in the expression of synapse-associated proteins, and dysfunction of the synaptic structure are closely related to cognitive dysfunction [33, 34]. To investigate whether *Erbin* knockout-induced cognitive impairment is related to the abovementioned changes, Nissl staining was used to evaluate CLP-induced neuronal lesions. As we expected, CLP mice displayed extensive damage to neurons in the CA1 and CA3 regions of the hippocampus, with pyknotic nuclei and cytoplasmic atrophy. Furthermore, more pyknotic and deep staining neurons were noticed in CA1 and CA3 areas in *Erbin*^−/−^ mice after CLP (Fig. 2A–C). We further observed synaptic ultrastructural pathological changes in the hippocampal CA1 region. TEM results revealed that the structure of synapses became blurred, presynaptic terminals were slightly swollen, and the thickness of the postsynaptic densities was decreased with widened synaptic clefts in septic mice.

Moreover, compared with the CLP group, thinner postsynaptic densities and broader synaptic clefts were observed in the *Erbin*^−/−^ + CLP group (Fig. 2D–F). At the same time, we examined PSD95, Synaptophysin (SYP), and Synapsin-1 (SYN1) expressions in the hippocampus by western blot and found PSD95, SYP, and SYN1 protein levels were dramatically down-regulated in mice underwent CLP operation. Not surprisingly, lower expressions of PSD95, SYP, and SYN1 were detected in *Erbin*^−/−^ septic mice (Fig. 2G–J). These results suggest that in septic mice, cognitive dysfunction aggravated by *Erbin* deletion is associated with neuronal damage, impaired synaptic structure, and reduced expression of synapse-related proteins in the hippocampus.

### *Erbin* deficiency facilitates NLRP3 inflammasome activation and pyroptosis of microglia in vivo and in vitro

**Fig. 3:**
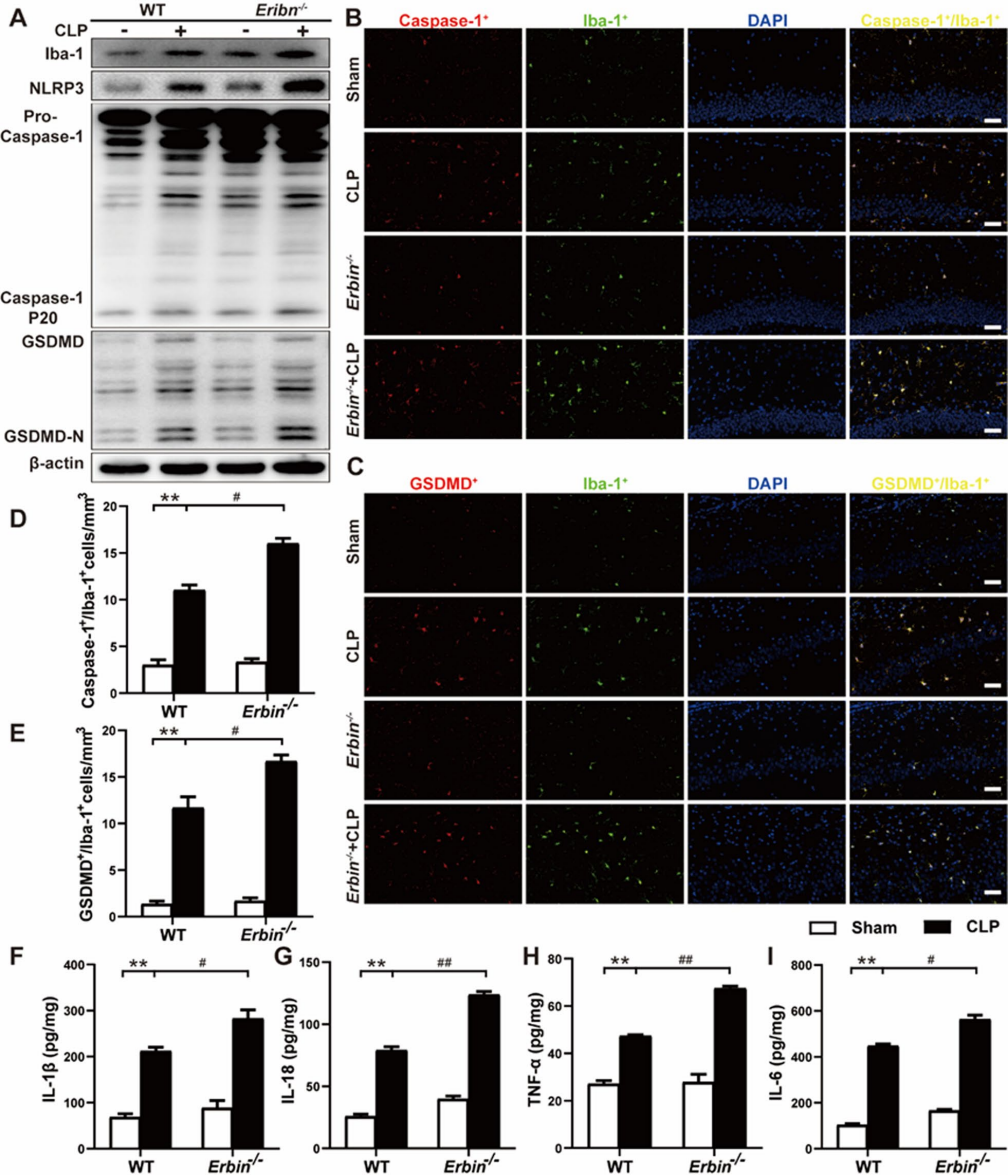
*Erbin* deficiency facilitates NLRP3 inflammasome activation and pyroptosis of microglia in vivo. **A** Protein levels of Iba-1, NLRP3, Caspase-1 P20, and GSDMD-N in the hippocampus were measured. **B-E** Numbers of Caspase-1/Iba1 and GSDMD/Iba1 positive cells in CA1 in the hippocampus were measured by double immunofluorescence staining. Scale bar, 50 μm. **F-I** IL-1β, IL-18, TNF-α, and IL-6 were measured in the hippocampus by ELISA. Data are representative of three independent experiments. *p < 0.05, **p < 0.01 vs. Sham group; ^#^*p* < 0.05, ^##^*p* < 0.01 vs. CLP group

Microglia-mediated neuroinflammation is proposed as a potential accelerator of neuronal injury and learning and memory decline in neuroinflammatory and neurodegenerative diseases [35]. We next examined the activation of microglia. As shown in Fig. 3A and Additional file 1: Fig. S1A, Iba-1 (activation markers of microglia) protein level significantly increased in septic mice, and *Erbin* knockout further elevated Iba-1 levels, indicating that *Erbin* deficiency exacerbated CLP-induced microglial activation. To further determine whether the activation of microglia is related to the NLRP3 inflammasome and pyroptosis, pyroptosis-associated proteins were detectable in mice hippocampus tissue 24 h after CLP, with increased NLRP3, Caspase-1 P20, and GSDMD-N protein levels in CLP group. Besides, *Erbin* knockout resulted in higher expression of these proteins in *Erbin*^−/−^ septic mice (Fig. 3A and Additional file 1: Fig. S1B–D). Meanwhile, coincident results were also obtained with double immunofluorescence staining. The number of Caspase-1/Iba-1 and GSDMD/Iba-1 double-positive cells in the CA1 area increased in septic mice, and Caspase-1^+^ and GSDMD^+^ cells increased consistent with Iba-1 localization (Fig. 3B–E), which suggested that pyroptosis mainly occurs in microglia. Moreover, the double-positive cells were further increased when *Erbin* was deleted. Subsequently, we tested inflammatory cytokines using ELISA kits and found that CLP operation effectively enhanced IL-1β, IL-18, TNF-α, and IL-6 levels in the hippocampus (Fig. 3F–I). While more inflammatory cytokines were detected in *Erbin*^−/−^ septic mice. Together, we conclude that NLRP3 inflammasome activation and pyroptosis in microglia were involved in CLP-induced SAE, which was further exacerbated by *Erbin* knockout.

**Fig. 4:**
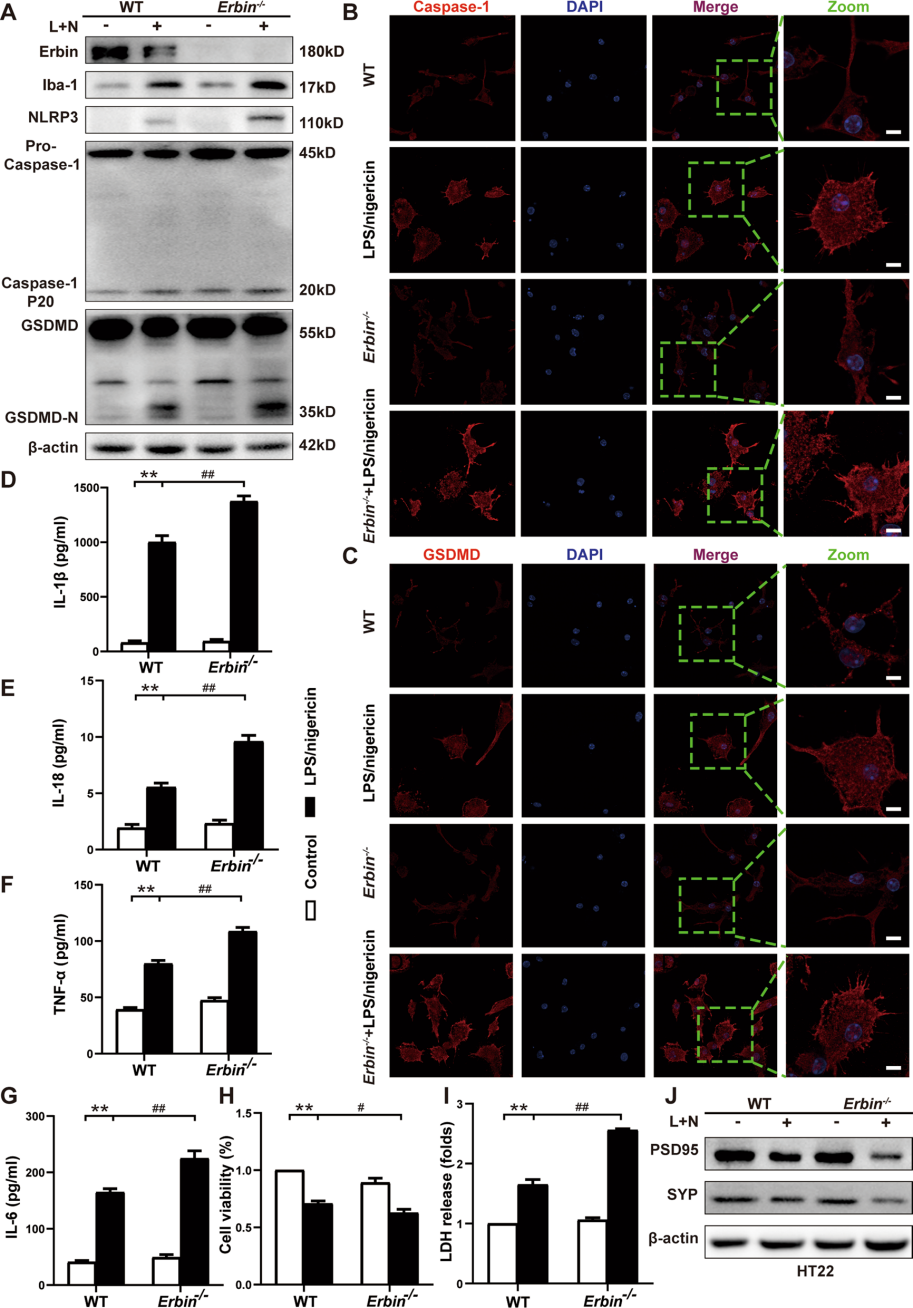
*Erbin* deficiency facilitates NLRP3 inflammasome activation and pyroptosis of microglia in primary microglia. **A** Protein levels of Erbin, Iba-1, NLRP3, Caspase-1 P20, and GSDMD-N in primary microglia were measured by western blot. **B**, **C** Fluorescence intensity and morphological changes of primary microglia were observed by confocal microscope. Scale bar, 8 μm. **D–G** IL-1β, IL-18, TNF-α, and IL-6 levels in primary microglia supernatant were measured by ELISA. **H** Cell viability of primary microglia was examined by CCK8. **I** Relative LDH release was detected by LDH kits in primary microglia. **J** Synapse-related proteins PSD95 and SYP in HT22 cells were measured by western blot. Data are representative of three independent experiments. *p < 0.05, **p < 0.01 vs. Control group; ^#^*p* < 0.05, ^##^*p* < 0.01 vs. LPS/nigericin group

Subsequently, we further verified this conclusion in vitro. We treated mouse primary microglia with LPS/nigericin to activate inflammation and found that levels of Erbin were down-regulated compared with controls following LPS/nigericin stimulation (Fig. 4A and Additional file 1: Fig. S1E). We assessed pyroptosis-related protein expression in primary microglia by western blot and immunofluorescence. LPS/nigericin treatment drastically increased the NLRP3 protein level and promoted Caspase-1 P20 and GSDMD-N expression in primary microglia derived from *Erbin*^−/−^ mice compared to controls (Fig. 4A and Additional file 1: Fig. S1G–I). Immunofluorescence results showed that activated primary microglia had enlarged cell bodies and reduced branches in typical amoeba-like changes after LPS/nigericin stimulation. In contrast, resting cells were rod-shaped or branched, which was consistent with the previously described resting and activated state of microglia [36]. The mean fluorescence intensity (MFI) analysis also showed that the MFI of Caspase-1 and GSDMD in *Erbin*^−/−^ cells was significantly higher than that of WT mice cells after LPS/nigericin stimulation (Fig. 4B, C).

Then, we detected protein levels of Iba-1 in primary microglia and inflammatory factors IL-1β, IL-18, TNF-α, and IL-6 in cultured supernatant cells. The results suggested that Iba-1 and inflammatory factors were significantly increased after LPS/nigericin treatment, and the above proteins were further increased after *Erbin* knockout (Fig. 4A, D–G). In addition, similar results were observed in BV2 cells with decreased Erbin expression and increased pyroptosis-related genes expression (NLRP3, Caspase-1 P20, and GSDMD-N) (Additional file 1: Fig. S1J, S1M–O) in the LPS/nigericin group, as well as protein levels of Iba-1 (Additional file 1: Fig. S1J, L) and mRNA levels of inflammatory factors (IL-1β, TNF-α, and IL-6) (Additional file 1: Fig. S1P–R). Furthermore, this change became more pronounced after *Erbin* knocked down with siRNA. CCK8 and LDH release assays, which could be used as an indicator to detect the occurrence of pyroptosis, also confirmed that *Erbin* knockout accelerated LPS/nigericin-stimulated microglia pyroptosis (Fig. 4H–I). Collectively, these results suggest that *Erbin* deficiency exacerbates microglia pyroptosis, and this effect may be relevant to the NLRP3 inflammasome.

To simulate the effect of microglia on hippocampal neurons in more detail in vivo, we proved influential findings by performing additional in vitro studies. Conditioned medium was collected from primary microglia generated from WT and *Erbin*^−/−^ mice after LPS priming and nigericin activation to induce pyroptosis and subsequently added to HT22 hippocampal neuron culture medium. Consistent with in vivo results, LPS/nigericin-treated cell supernatants significantly reduced cell viability (Additional file 1: Fig. S1S) and the expression of PSD95 and SYP in HT22 cells (Fig. 4J). In comparison, *Erbin* loss resulted in lower cell viability and synapse-associated protein levels.

### Erbin regulates Ca^2+^ mobilization via IRE1α/Xbp1s pathway in microglia

**Fig. 5:**
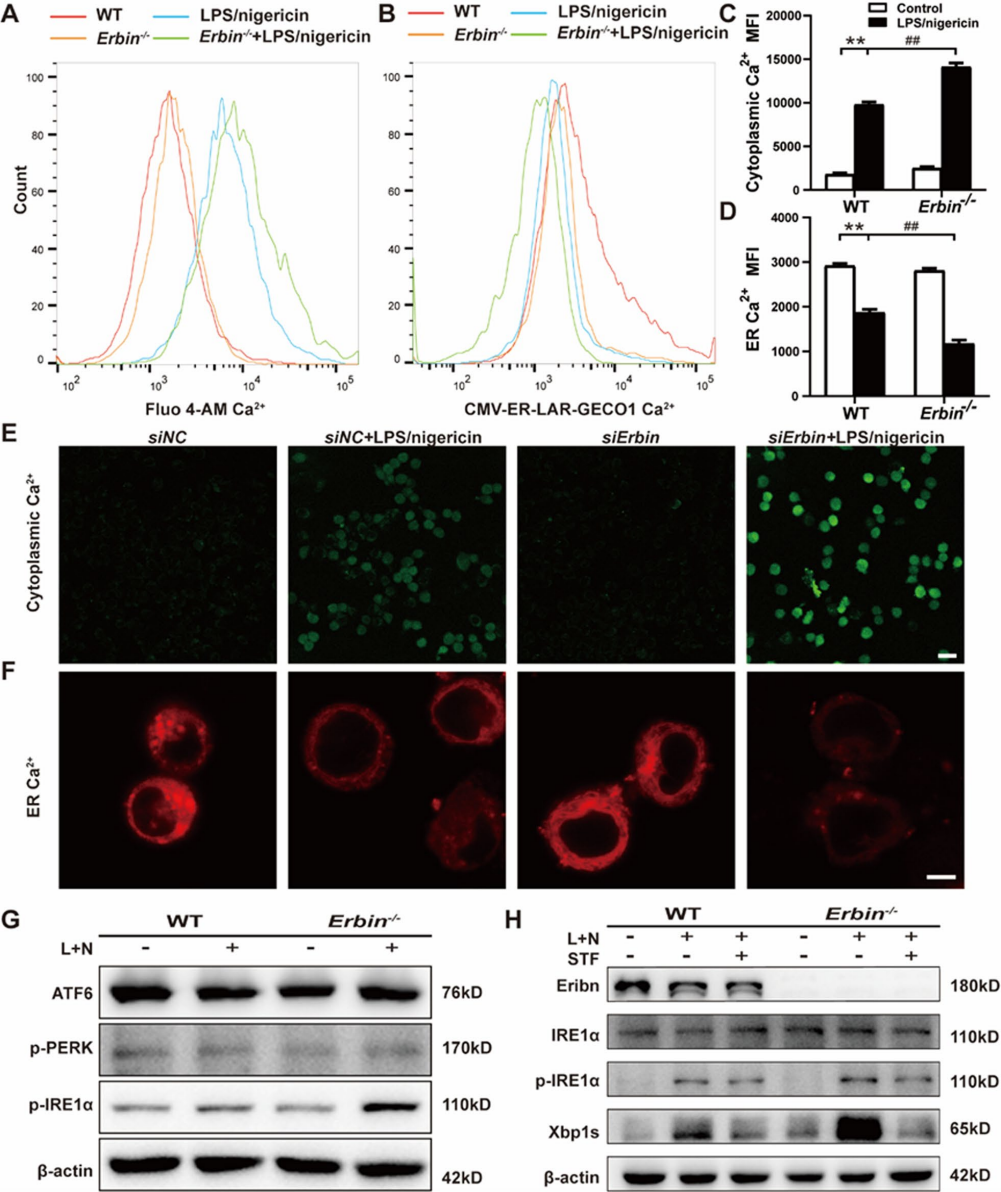
Erbin regulates Ca^2+^ mobilization in primary microglia and BV2 cells. **A** Fluo4-AM was used to indicate the changes of intracytoplasmic Ca^2+^ of primary microglia by flow cytometry. **B** ER Ca^2+^ of primary microglia located by ER-targeted plasmid CMV-ER-LAR-GECO1 were observed by flow cytometry. **C**, **D** MFI of intracytoplasmic Ca^2+^ and ER Ca^2+^ in primary microglia were analyzed by flow cytometry. **E** Concentrations of intracytoplasmic Ca^2+^ in BV2 cells were observed by confocal microscopy. Scale bar, 25 μm. **F** Concentrations of ER Ca^2+^ in BV2 cells were observed by confocal microscopy. Scale bar, 5 μm. **G** Three main ER stress sensors, ATF6, PERK, and IRE1α, were measured by western blot in primary microglia. **H** Added STF083010, expressions of Erbin, IRE1α, p-IRE1α and Xbp1s in primary microglia were analyzed by western blot. Data are representative of three independent experiments. *p < 0.05, **p < 0.01 vs. Control group; ^#^*p* < 0.05, ^##^*p* < 0.01 vs. LPS/nigericin group

Ca^2+^ mobilization is thought to be an essential trigger for NLRP3 inflammasome activation [37]. We used Fluo-4 AM Ca^2+^ probes to detect intracellular Ca^2+^, and MFI was recorded by flow cytometry. Flow cytometry results demonstrated that intracellular Ca^2+^ accumulation was dramatically upregulated by treatment with LPS/nigericin, which was further enhanced in the absence of *Erbin* in primary microglia (Fig. 5A, C). Therefore, we speculated that Erbin suppressed pyroptosis by reducing cytoplasmic Ca^2+^ accumulation. Ca^2+^ mobilization occurs by releasing ER-linked intracellular Ca^2+^ pools or opening plasma membrane channels, allowing Ca^2+^ flow to the cytoplasm. As the major intracellular Ca^2+^ storage organelle, ER regulates the flow of Ca^2+^ under stress conditions. Using ER-targeted plasmid CMV-ER-LAR-GECO1, a sensitive single-wavelength Ca^2+^ indicator for detecting Ca^2+^ dynamics within the ER, we found ER Ca^2+^ was apparently reduced after LPS/nigericin administration, which was further decreased in *Erbin*^−/−^ primary microglia (Fig. 5B, D). Confocal results in BV2 cells showed that intracellular Ca^2+^ only displayed a weak green light, and the green light instantly brightened when LPS/nigericin was added. Just as *Erbin* was silenced, the brightness was further enhanced in the *siErbin* + LPS/nigericin group compared with the *siNC* + LPS/nigericin group (Fig. 5E). In contrast, the Ca^2+^ in the ER changed precisely opposite to the trend of intracytoplasmic Ca^2+^ (Fig. 5F). All these results suggest that the increased cytoplasmic Ca^2+^ may come, at least in part, from ER.

LPS stimulation can cause ER stress. Three unfolded protein sensors, IRE1α, PERK, and ATF6, reside in ER and are involved in regulating ER stress and ER Ca^2+^ release [38, 39]. To determine whether UPR signaling was activated during inflammasome formation, we detected the activation of IRE1α, PERK, and ATF6 in primary microglia after LPS/nigericin treatment. Consistent with previous reports, activation of IRE1α was discovered in cell lysates, reflected in increased p-IRE1α protein levels. PERK or ATF6 branches were not detected following LPS/nigericin treatment by western blot (Fig. 5G and Additional file 1: Fig. S2A–C). More importantly, *Erbin* knockout facilitated IRE1α phosphorylation within LPS/nigericin-stimulated conditions, indicating that the expression of Erbin is negatively correlated with the activation of IRE1α.

**Fig. 6:**
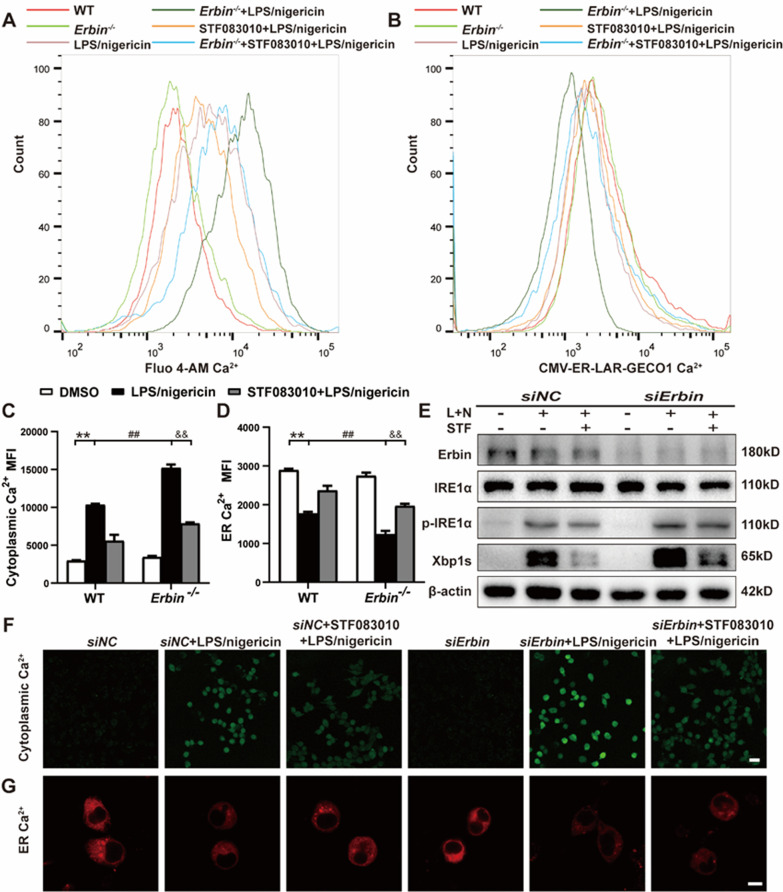
Erbin regulates Ca^2+^ mobilization via IRE1α/Xbp1s pathway in primary microglia and BV2 cells. **A** Concentrations of intracytoplasmic Ca^2+^ of primary microglia after being given STF083010 were measured by flow cytometry. **B** ER Ca^2+^ of primary microglia after given STF083010 were observed by flow cytometry. **C**, **D** MFI of intracytoplasmic Ca^2+^ and ER Ca^2+^ in primary microglia were analyzed by flow cytometry. **E** Erbin and IRE1α/Xbp1s pathways proteins expression in BV2 cells were analyzed by western blot. **F** Intracytoplasmic Ca^2+^ concentrations were observed by confocal microscopy. Scale bar, 25 μm. **G** ER Ca^2+^ concentrations were observed through confocal microscopy. Data are representative of three independent experiments. Scale bar, 8 μm. *p < 0.05, **p < 0.01 vs. Control group; ^#^*p* < 0.05, ^##^*p* < 0.01 vs. LPS/nigericin group; ^&^*p* < 0.05, ^*&&*^*p* < 0.01 vs. *Erbin*^*−/−*^ + LPS/nigericin group

To investigate whether increased cytoplasmic Ca^2+^ was associated with IRE1α signaling, we utilized STF083010, a selective inhibitor of IRE1α RNase activity, to block the IRE1α signaling and observed the changes in Ca^2+^ concentration in the cytoplasm and ER. STF083010 significantly inhibited IRE1α signaling, as reflected in decreased p-IRE1α and Xbp1s expressions (Fig. 5H and Additional file 1: Fig. S2E–F). However, the addition of STF083010 did not affect the expression of Erbin (Fig. 5H and Additional file 1: Fig. S2D), suggesting that Erbin should be located upstream of IRE1α/Xbp1s pathway. Although *Erbin* deficiency enhanced the activity of the IRE1α/Xbp1s pathway after LPS/nigericin treatment, the addition of STF083010 partially counteracted this activation. Not only the expression of IRE1α and Xbp1s protein was reduced, but also accompanied by a decrease in cytoplasmic Ca^2+^ in primary microglia detected by the flow cytometer in the *Erbin*^−/−^ + STF083010 + LPS/nigericin group (Fig. 6A, C). Besides, ER Ca^2+^ was partially restored after given STF083010 (Fig. 6B, D). In BV2 cells, we also observed that STF083010 could inhibit IRE1α/Xbp1s pathway activity which is activated by LPS/nigericin stimulation under the condition of *Erbin* silence (Fig. 6E and Additional file 1: Fig. S2G–I). Subsequent confocal assays also confirmed this conclusion (Fig. 6F, G). With the addition of STF083010, the fluorescence intensity of intracellular Ca^2+^ was weakened, and that of ER Ca^2+^ was relatively increased in BV2 cells which transfected with *siErbin*. Altogether, these results demonstrated that Erbin restrained Ca^2+^ flow from ER to the cytoplasm via inhibiting IRE1α/Xbp1s pathway under LPS/nigericin-stimulated conditions.

### Erbin regulates pyroptosis via IRE1α/Xbp1s-Ca^2+^ axis in vivo and in vitro

**Fig. 7:**
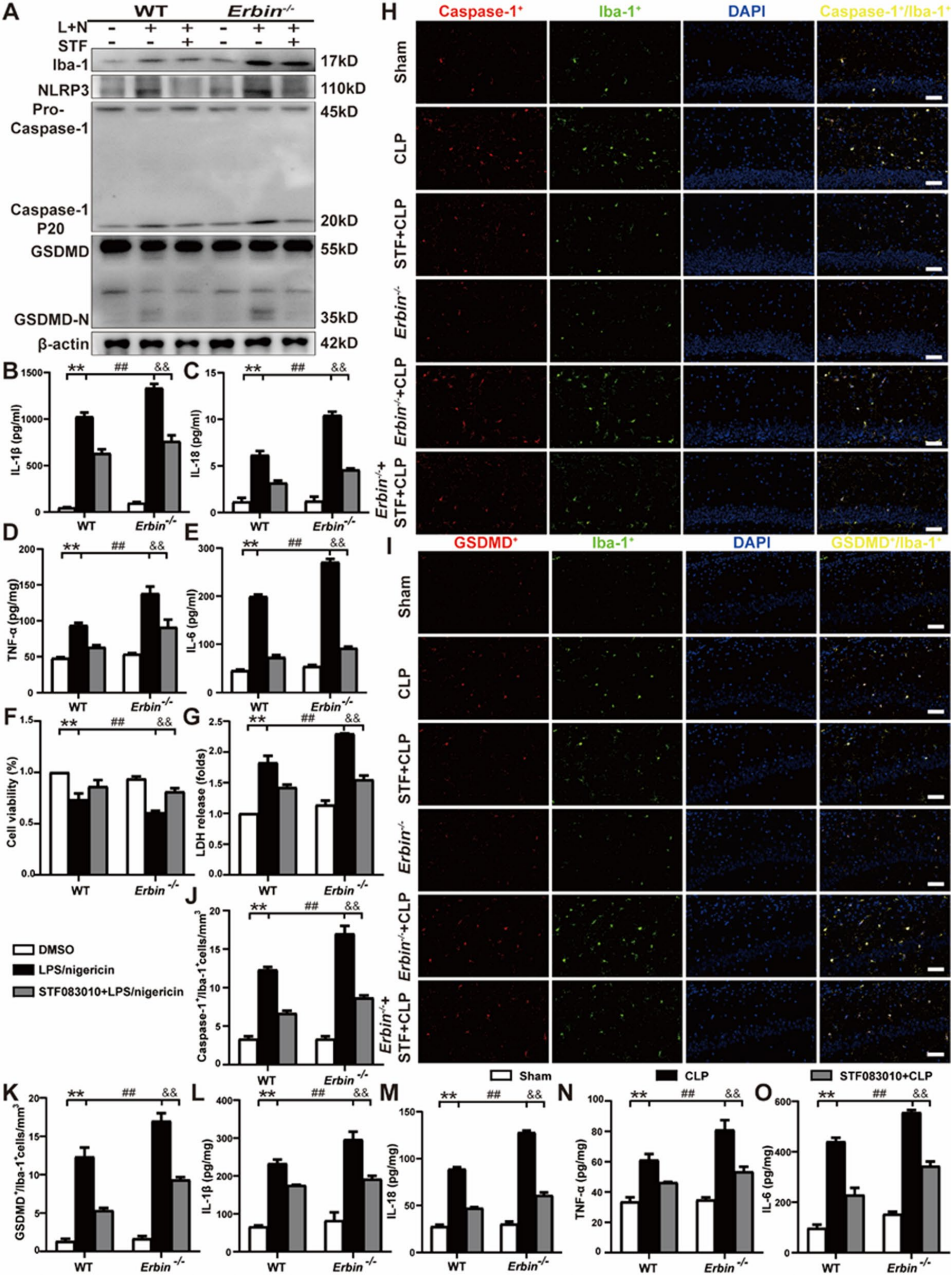
Erbin regulates pyroptosis via IRE1α/Xbp1s/Ca^2+^ in vivo and in vitro. **A** Western blot for pyroptosis-related proteins in primary microglia. **B–E** IL-1β, IL-18, TNF-α, and IL-6 release in the supernatants of primary microglia were measured using the ELISA method. **F–G** Cell viability and relative LDH release were detected by CCK8 and LDH kits in primary microglia. **H–K** Double immunofluorescence staining was used to observe the numbers of Caspase-1/Iba1 and GSDMD/Iba1 positive cells in the CA1 area of the hippocampus. Scale bar, 50 μm. **L–O** IL-1β, IL-18, TNF-α, and IL-6 levels were measured in the hippocampus by ELISA. Data are representative of three independent experiments. *p < 0.05, **p < 0.01 vs. Control or Sham group; ^#^*p* < 0.05, ^##^*p* < 0.01 vs. LPS/nigericin or CLP group; ^&^*p* < 0.05, ^*&&*^*p* < 0.01 vs. *Erbin*^*−/−*^ + LPS/nigericin or *Erbin*^*−/−*^ + CLP group

Since Erbin could regulate ER Ca^2+^ mobilization through IRE1α/Xbp1s, we speculated that Erbin could also inhibit NLRP3 inflammasome and pyroptosis by IRE1α/Xbp1s-Ca^2+^ axis. To verify this, we detected pyroptosis-related proteins levels and inflammatory cytokines in the case of blocking IRE1α RNase activity in primary microglia. After adding STF083010 into primary microglia derived from *Erbin*^−/−^ mice, we observed the expression of pyroptosis-related proteins was also conspicuously decreased, behaved as diminished protein expression of Iba-1, NLRP3, Caspase-1 P20, GSDMD-N (Fig. 7A and Additional file 1: Fig. S2J–M) and reduced release of inflammatory factors (IL-1β, IL-18, TNF-α, and IL-6) compared with the *Erbin*^−/−^ + LPS/nigericin group (Fig. 7B–E). Simultaneously, we also verified our hypothesis on BV2 cells. As we expected, STF083010 markedly suppressed Iba-1, NLRP3, Caspase-1 P20, GSDMD-N protein expressions (Additional file 1: Fig. S3A–E), and IL-1β, TNF-α, and IL-6 mRNA levels in the *siErbin* + STF083010 + LPS/nigericin group compared with the *siErbin* + LPS/nigericin group (Additional file 1: Fig. S3F–H). In addition, we tested the cell viability and the LDH release of primary microglia. The addition of STF083010 ameliorated the onset of pyroptosis in LPS/nigericin treated *Erbin*^−/−^ cells, manifested as increased cell viability and decreased LDH release (Fig. 7F–G). We also validated it in animals using immunofluorescence. As shown in Fig. 7H–K, the number of Caspase-1/Iba1 and GSDMD/Iba1 double-positive cells were reduced, and IL-1β, IL-18, TNF-α, and IL-6 levels were decreased with STF083010 administration in *Erbin*^−/−^ septic mice (Fig. 7L–O).

Combined with previous results, knockout of *Erbin* weakened its inhibition on the IRE1α/Xbp1s pathway, enhanced its activity, mobilized ER Ca^2+^ to flow to the cytoplasm, and consequently led to the activation of NLRP3 inflammasome and occurrence of pyroptosis. In conclusion, Erbin negatively modulates microglia pyroptosis through IRE1α/Xbp1s-Ca^2+^ pathway.

### Erbin alleviates neuronal damage and improves cognitive function by inhibiting IRE1α/Xbp1s pathway

**Fig. 8:**
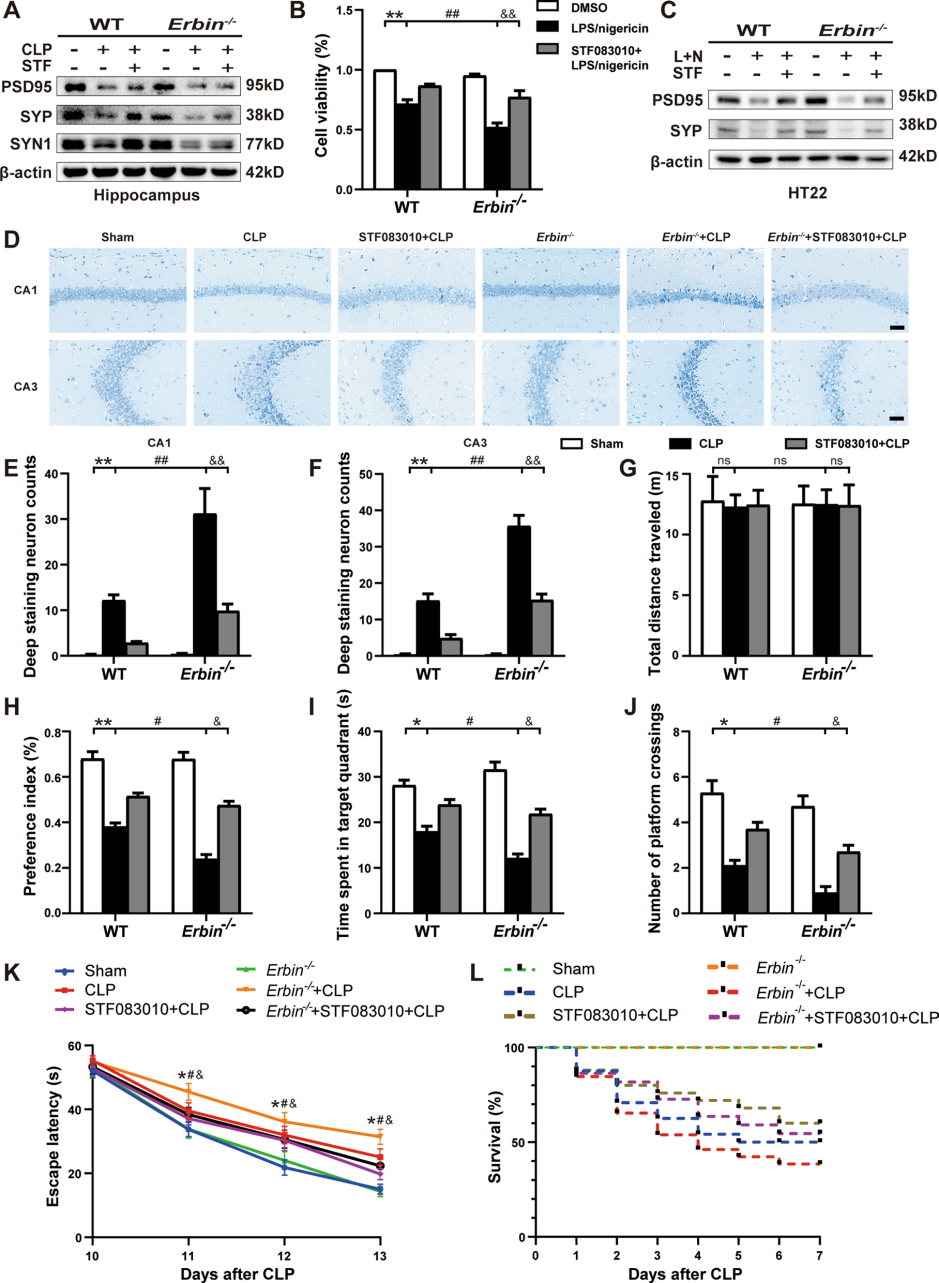
Erbin alleviates neuronal damage and improves cognitive function by inhibiting IRE1α/Xbp1s pathway. **A** Western blot for PSD95, SYP, and SYN1 protein expressions in the hippocampus. **B** Cell viability of HT22 cells cultured in microglia CM was examined by CCK8. **C** Western blot for PSD95 and SYP protein expressions in microglia CM cultured HT22 cells. **D-F** Neuronal damage was measured by Nissl staining. Scale bar, 50 μm. **G** Traveled distance, **H** preference indices, **I** time spent in the target quadrant, **J** numbers of crossings through the platform, and **K** escapes latency was measured in each group (n = 10). **L** 7-day survival rate of mice was recorded after CLP. Data are representative of three independent experiments. *p < 0.05, **p < 0.01 vs. Sham group; ^#^*p* < 0.05, ^##^*p* < 0.01 vs. CLP group; ^&^*p* < 0.05, ^*&&*^*p* < 0.01 vs. *Erbin*^*−/−*^ + CLP group

**Fig. 9:**
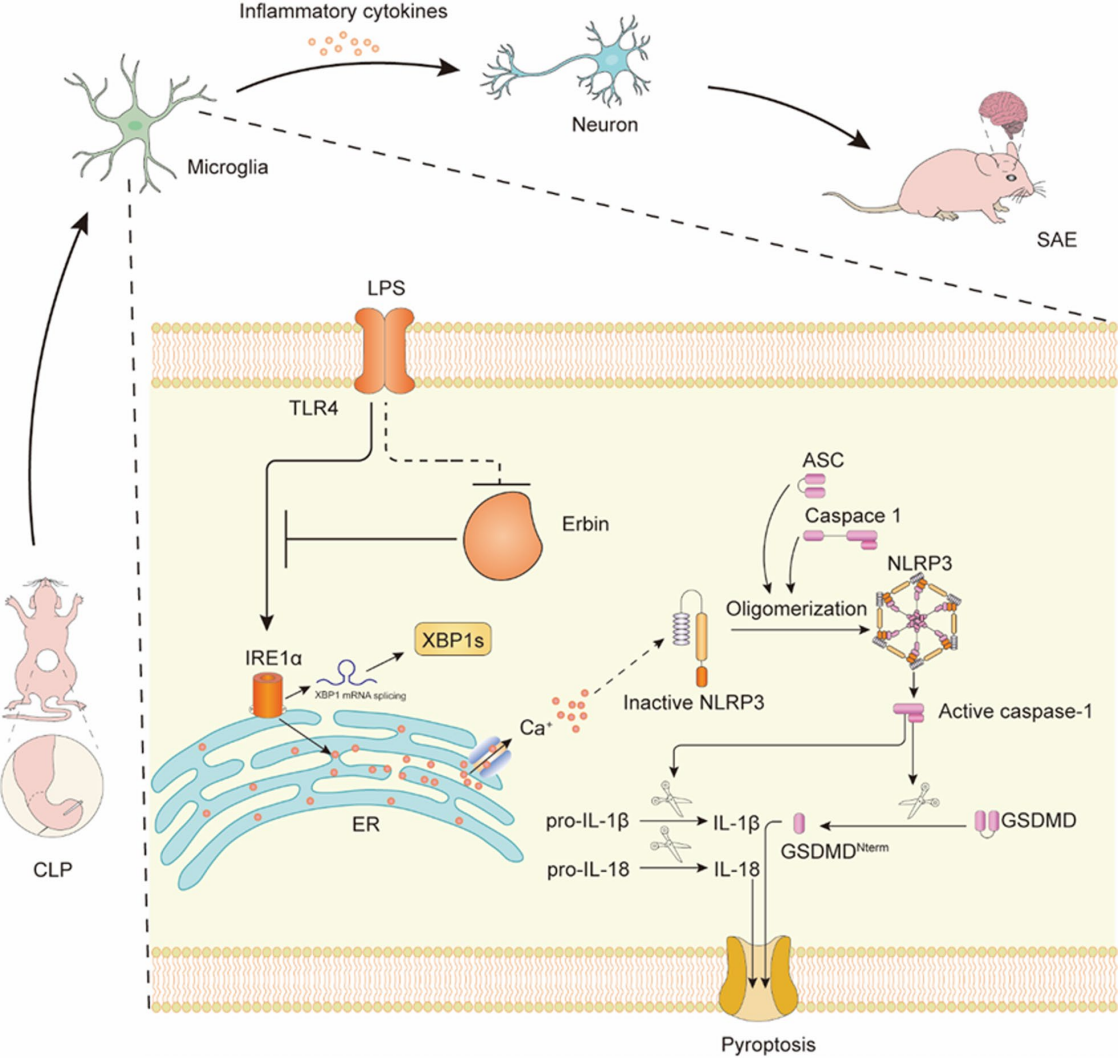
Erbin plays a protective role in SAE. CLP-induced sepsis can activate microglia in the brain. Under the stimulation of LPS, the expression of Erbin is down-regulated, which accelerates the activation of the IRE1α/Xbp1s pathway, which induces the transfer of Ca^2+^ from ER to cytoplasm, and triggers the activation of downstream NLRP3 inflammasomes and the occurrence of pyroptosis, leading to the release of a large number of inflammatory cytokines, causing neuronal damage and synaptic structure damage in the hippocampus, finally resulting in cognitive dysfunction

We next examined the alterations of synaptic protein levels. As we speculated, STF083010 significantly elevated PSD95, SYP, and SYN1 protein expressions in the hippocampus in *Erbin*^−/−^ septic mice (Fig. 8A and Additional file 1: Fig. S3I–K). Moreover, similar results were obtained in HT22 cells. When HT22 cells were cultured with STF083010-treated *Erbin*^−/−^ microglia supernatant, the protein levels of PSD95 and SYP were partially restored to control levels (Fig. 8C and Additional file 1: Fig. S3L–M), accompanied by an increase in cell viability (Fig. 8B). Compared with the *Erbin*^−/−^ + CLP group, decreased pyknotic neurons and increased normal morphological neurons in CA1 and CA3 regions were observed after the mice were given STF083010 (Fig. 8D–F). Subsequently, the behavioral performance of mice was explored and higher recognition index (Fig. 8H), shorter latencies in 2–4 days (Fig. 8K), more extended time in the target quadrant (Fig. 8I), and more times across platforms (Fig. 8J) were observed after STF083010 treatment in *Erbin*^−/−^ septic mice. This might be attributable to the fact that STF083010 specifically blocked the activity of the IRE1α/Xbp1s pathway induced by CLP, which was further enhanced after *Erbin* knockout, reduced microglia pyroptosis and inflammatory factors release, thereby alleviating neuronal damage and increasing the expression of synapse-related proteins, ultimately improving cognitive dysfunction. In addition, the high mortality caused by *Erbin* deficiency (survival rate 38.5%, 10 of 26 mice survived) was ameliorated by giving STF083010 (survival rate 54.5%, 12 of 22 mice survived) in SAE mice (Figs. 8L, 9).

All the above results suggested that, once *Erbin* was knocked out in microglia, its negative regulatory effect on IRE1α/Xbp1s was attenuated, resulting in a relative activation of IRE1α/Xbp1s, thereby activating NLRP3 inflammasome, causing microglia pyroptosis, which released mounts of inflammatory factors, leading to neuronal damage and even cognitive impairment in sepsis mice.

## Discussion

Erbin is involved in inhibiting various cell signaling pathways as a scaffold protein. Erbin plays a cardioprotective role in cardiovascular disease, and the down-regulation of Erbin is associated with cardiac hypertrophy and heart failure in both mice and humans [40]. A few research reports showed that Erbin exerted a protective effect against inflammatory bowel disease by suppressing autophagic cell death, and *Erbin*^−/−^ mice were more prone to small intestinal inflammation [27]. Though Erbin is highly expressed in the brain, its role in CNS in SAE mice has not been explored. In this study, we discovered the neuroprotective effects of Erbin in SAE by regulating IRE1α/Xbp1s-Ca^2+^ axis and consequently inhibiting microglia pyroptosis.

SAE is a common and severe complication of sepsis and is closely associated with mortality and morbidity. Several studies have shown an association between brain injury and long-term psychological or cognitive impairment in SAE [41, 42]. Consistent with previous studies, septic mice exhibited severe learning and memory impairment 7 days after CLP assessed by behavioral tests. Lack of Erbin exacerbated cognitive impairment and accelerated death in septic mice. More importantly, Erbin was down-regulated in septic mice. Although we cannot conclude that this downregulation is a cause of SAE rather than its consequence, it is a reasonable hypothesis based on our results in mice. Thus, for the first time, this study shows the role of Erbin in SAE and suggests that it is crucial in attenuating cognitive dysfunction induced by CLP.

The hippocampus is one of the brain structures that is more sensitive to the cognitive dysfunction caused by sepsis [43]. Damage to hippocampal CA1 and CA3 regions, critical brain regions for learning and memory, may lead to cognitive impairment [44]. Our study observed more severe neuronal damage in the CA1 and CA3 areas in *Erbin*^−/−^ septic mice. Dysregulation of synaptic ultrastructure is associated with cognitive impairment [45, 46]. Aberrant alterations in the morphological structure of postsynaptic densities may result in long-term synaptic plasticity impairment and synaptic transmission dysfunction [47]. Subsequently, the TEM result showed that the synaptic structure was deteriorated with thinner postsynaptic densities and broader synaptic clefts in *Erbin*^−/−^ septic mice. Synapse-related proteins, including SYP, SYN1, and PSD95, play crucial roles in synaptic plasticity and memory formation [48]. SYP is present in neuroendocrine cells and implicated in synaptic transmission. Low expression of SYP led to behavioral changes, such as difficulty in object novelty recognition and reduction in spatial learning [49]. SYN1 exists in the nerve terminal of axons and is involved in the regulation of synaptogenesis and neurotransmitter release. PSD95, a protein density, is almost completely attached to the postsynaptic membrane, participates in anchoring synaptic proteins, regulating synaptic plasticity, and plays an important role in long-term potentiation [50]. Here, *Erbin* deletion in septic animals further downregulated synapse-related protein levels compared with the CLP group. Therefore, it is speculative that Erbin ameliorates cognitive dysfunction by reducing neuronal damage, improving the synaptic structure, and increasing synapse-associated protein expressions.

Systemic inflammation during sepsis could activate microglia in the brain, then activated microglia, and following secreted pro-inflammatory cytokines are detrimental to neuronal survival, growth, synaptogenesis, and phagocytosis [51]. Aberrant activation of NLRP3 inflammasome and pyroptosis has been described as dominant factors in microglia-mediated neuroinflammation in SAE development [7]. Once NLRP3 inflammasome activated, pro-Caspase-1 was cleaved into active Caspase-1, then activated Caspase-1 cleaved the GSDMD, pro-IL-1β and pro-IL-18, leading to pyroptosis and IL-1β and IL-18 secretion. IL-1β should be the main perpetrator of neurogenesis. Studies have shown that neurons highly express IL-1 receptors, and excessive IL-1β can significantly inhibit the proliferation and growth of neurons [52]. Overexpression of IL-1β in the hippocampus may affect neuronal synaptic plasticity and impairs hippocampal-dependent memory [53]. Treated with MCC950 (the NLRP3 inhibitor) [7] or Ac-YVAD-CMK (the Caspase-1 inhibitor) [6] prevented sepsis-induced neuronal damage and cognitive deficits. To investigate whether the effect of Erbin is related to pyroptosis-mediated neuroinflammation in microglia, we systematically examined the activation of microglia and the expression of pyroptosis-associated genes and found that Iba-1 and pyroptosis-associated proteins were all overexpressed in hippocampus tissue after CLP. What is more, the increase in the above indicators was more pronounced in *Erbin*^−/−^ septic mice, as well as increased pro-inflammatory cytokine levels. We also observed intense Caspase-1 and GSDMD immunostaining in Iba-1-labeled microglia and remarkable growth in hippocampal lesions in *Erbin*^−/−^ mice. Collectively, the present results define that the pyroptosis occurring in microglia can be inhibited by Erbin in SAE. We further verified the conclusion in primary mouse microglia and BV2 cells. Afterward, the viability of HT22 cells cultured with CM of LPS/nigericin-treated *Erbin*-knockout microglia was obviously reduced. This suggested that *Erbin*'s deletion exacerbated neuronal damage and cognitive dysfunction due to the overactivation of NLRP3 inflammasome and pyroptosis.

Ca^2+^ mobilization is an essential upstream event for NLRP3 inflammasome activation. It occurs by opening plasma membrane channels or releasing ER-linked intracellular Ca^2+^ stores to allow Ca^2+^ to flow into the cytosol [54]. Inhibition of Ca^2+^ mobilization has been reported to reduce the activation of the NLRP3 inflammasome [37, 55]. Our study detected a pronounced accumulation of intracytoplasmic Ca^2+^ in response to LPS/nigericin stimulation, and a further increase in intracytoplasmic Ca^2+^ was observed when *Erbin* was knockout or knockdown, indicating that Erbin might have a negative effect on the regulation of Ca^2+^ in the cytoplasm. Given that ER is the most important intracellular Ca^2+^ store and increased intracellular Ca^2+^ influx from the ER is an endogenous signal which drives cell death and immune responses [56], we continued to explore whether the increased cytosolic Ca^2+^ came, at least in part, from the ER. Results of flow cytometry and confocal microscopy both showed that Ca^2+^ in ER was significantly reduced after LPS/nigericin administration. ER stress is closely linked to dysregulated Ca^2+^ homeostasis, and three ER stress signaling is involved in regulating ER Ca^2+^ release [38, 39]. We then found only IRE1α was phosphorylated upon LPS stimulation, accompanied by increased splicing of downstream XBP1. In agreement with previous works, toll-like receptors (TLR), which primarily recognize microbial ligands like LPS, selectively stimulate IRE1α, but not PERK or ATF6 [57]. Also, IRE1α/XBP1 activated by TLR is essential for optimal and sustained production of pro-inflammatory cytokines in macrophages [57]. Therefore, we speculate that the activation of the IRE1α/Xbp1s signaling may be related to the increase of intracytoplasmic Ca^2+^ and the decrease of ER Ca^2+^. Furthermore, *Erbin* depletion increased the expression of p-IRE1α and Xbp1s, indicating that Erbin negatively modulates IRE1α/Xbp1s pathways in LPS/nigericin-treated microglia and CLP-induced SAE mice.

The movement of Ca^2+^ across the ER membrane is mediated by Ca^2+^ release channels, including inositol-1,4,5-triphosphate receptors (InsP3Rs) and ryanodine receptors (RyRs). Some studies have demonstrated that IRE1α regulates the flow of Ca^2+^ between ER and mitochondria by determining the distribution of InsP3Rs located in ER membrane in mouse liver [58]. Also, Xbp1s could induce the expression of InsP3Rs, facilitating Ca^2+^ release from ER [59]. However, whether IRE1α or Xbp1s can regulate ER Ca^2+^ movement in microglia in SAE is unknown. To verify this, we blocked the IRE1α/Xbp1s pathway using STF083010, a specific inhibitor of IRE1α RNase activity inhibitor. Just as we envisioned, Ca^2+^ in the cytoplasm decreased, and Ca^2+^ in ER increased in LPS/nigericin-treated *Erbin*-silenced primary microglia or BV2 cells after being given STF083010. This suggests that Erbin can limit the efflux of Ca^2+^ from ER to cytoplasm by inhibiting IRE1α/Xbp1s pathway activity. Meanwhile, pyroptosis-associated proteins were all reduced both in *Erbin*-deficient primary microglia and BV2 cells after blocking IRE1α/Xbp1s signaling. The number of Caspase-1/Iba-1 and GSDMD/Iba-1 double-positive cells in the *Erbin*^−/−^ + CLP + STF083010 group was less than that in the *Erbin*^−/−^ + CLP group, as well as descending inflammatory cytokines levels. Moreover, the addition of STF083010 did not affect Erbin's expression. Combining the previous results, we can conclude that Erbin negatively regulates IRE1α/Xbp1s pathway in LPS/nigericin-treated microglia or CLP-induced SAE mice. When LPS added or sepsis occurs, the expression of Erbin decreases and the activity of the IRE1α/Xbp1s pathway is enhanced, mobilizing Ca^2+^ to flow from ER to cytoplasm, resulting in Ca^2+^ accumulation in the cytoplasm, thereby inducing inflammasome activation and pyroptosis, release inflammatory factors.

Finally, we assessed neurological impairment and behavioral performance in SAE mice followed by the administration of STF083010. As expected, STF083010 significantly attenuated neuronal damage in hippocampal increased pyroptosis-associated proteins expressions and improved cognitive dysfunction in *Erbin*^−/−^ septic mice. These results further support the idea that Erbin exerts neuroprotective effects by inhibiting the IRE1α/Xbp1s pathway in SAE mice.

ER stress-related TXNIP/NLRP3 inflammasome activation is involved in the pathophysiology of SAH [60], neonatal hypoxic-ischemic brain injury [20], type 2 diabetes [61], and many other diseases. Hyperactivated IRE1α activates the NLRP3 inflammasome through elevated TXNIP protein expression, resulting in caspase-1 cleavage and IL-1β secretion. TXNIP knockout reduces pyroptosis during ER stress. Small molecule IRE1α RNase inhibitors STF083010 inhibit TXNIP production from blocking IL-1β secretion. In our study, we also detected TNXIP expression in the hippocampus and microglia and found that LPS treatment resulted in decreased TXNIP expression (see Additional file 1: Fig. S4). Nevertheless, we did not find that IRE1α RNase signaling promotes inflammasome formation by increasing TXNIP expression.

However, some limitations in our study cannot be ignored. Firstly, the increased Ca^2+^ in the cytoplasm may come from the efflux of Ca^2+^ in intracellular organelles or from the influx of extracellular Ca^2+^. We did not further explore whether other organelles such as mitochondria and lysosomes have Ca^2+^ efflux into the cytoplasm to lead to NLRP3 inflammasome activation, nor did we completely rule out the effect of extracellular Ca^2+^ influx on NLRP3 inflammasome and pyroptosis. Secondly, we have not yet elucidated the exact mechanism of how the IRE1α/Xbp1s pathway regulates ER Ca^2+^ efflux and whether IRE1α/Xbp1s regulates ER Ca^2+^ efflux by affecting the Ca^2+^ channel InsP3Rs on the ER. Therefore, although the present study is helpful in understanding the neuroprotective role of Erbin in SAE, further experimental verification is still needed.

## Conclusion

In the current study, we first demonstrated that Erbin played a protective role in SAE, partly attributable to its inhibiting NLRP3 inflammasomes and pyroptosis via inhibiting IRE1α/Xbp1s-Ca^2+^ signaling. Through this mechanism, Erbin could diminish microglia activation and the inflammatory response it mediated, attenuated the damage to hippocampal neurons and ultimately improved cognitive dysfunction.

## Supplementary Information

**Additional file 1. Fig. S1. **Erbin deficiency facilitates NLRP3 inflammasome activation and pyroptosis of microglia in vivo and vitro.** Fig. S2. **Erbin regulates pyroptosis via IRE1α/Xbp1s/Ca2+ in vivo and vitro.** Fig. S3. **Erbin inhibits pyroptosis via IRE1α/Xbp1s/Ca2+ in BV2 cells and improves synaptic proteins by inhibiting IRE1α/Xbp1s pathway. **Fig. S4. **LPS/nigericin stimulation resulted in decreased TXNIP protein levels regardless of Erbin knockdown in BV2 cells.

## Data Availability

The data supporting the findings of this study are presented within the manuscript.

## Abbreviations

SAE: Sepsis-associated encephalopathy
ER: Endoplasmic reticulum
CNS: Central nervous system
GSDMD-N: N-terminal fragment of GSDMD
IRE1α: Inositol requiring enzyme-1 alpha
PERK: PKR-like endoplasmic reticulum kinase
ATF-6: Activating transcription factor-6
XBP1: X-box-binding protein 1
XBP1s: Spliced XBP1
CLP: Cecal ligation and puncture
OFT: Open field test
NORT: Novel object recognition test
MWM: Morris Water Maze
CM: Conditioned medium
RT-qPCR: Reverse transcription-quantitative real-time polymerase chain reaction
TEM: Transmission electron microscopy
PSD95: Postsynaptic density 95
SYP: Synaptophysin
SYN1: Synapsin-1
InsP3Rs: Inositol-1,4,5-triphosphate receptors
