# Supplementary material for: Erbin protects against sepsis-associated encephalopathy by attenuating microglia pyroptosis via IRE1α/Xbp1s-Ca2+ axis
Source: J Neuroinflammation. 2022 Sep 28;19:237. doi: 10.1186/s12974-022-02598-5 (PMC9520943; doi:10.1186/s12974-022-02598-5)
Supplement: Supplementary file 1 — Additional file 1. Fig. S1. Erbin deficiency facilitates NLRP3 inflammasome activation and pyroptosis of microglia in vivo and vitro. Fig. S2. Erbin regulates pyroptosis via IRE1α/Xbp1s/Ca2+ in vivo and vitro. Fig. S3. Erbin inhibits pyroptosis via IRE1α/Xbp1s/Ca2+ in BV2 cells and improves synaptic proteins by inhibiting IRE1α/Xbp1s pathway. Fig. S4. LPS/nigericin stimulation resulted in decreased TXNIP protein levels regardless of Erbin knockdown in BV2 cells. [file 12974_2022_2598_MOESM1_ESM.docx]

**Additional file**


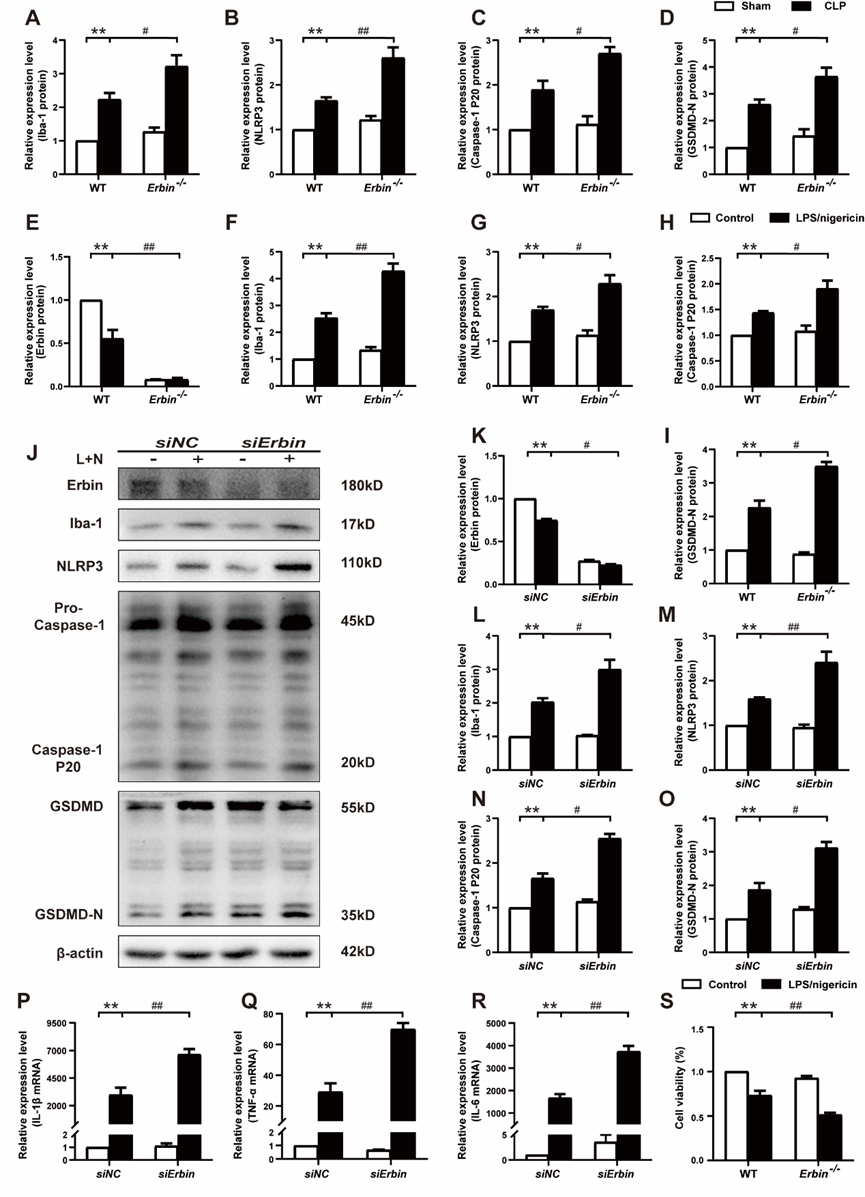


**Fig.S1. Erbin deficiency facilitates NLRP3 inflammasome activation and pyroptosis of microglia in vivo and vitro.**

**A-D** Western blot was used to estimate the protein levels of Iba-1, NLRP3, Caspase-1 P20 and GSDMD-N in hippocampus. **E-I** Western blot was used to estimate the protein levels of Erbin, Iba-1, NLRP3, Caspase-1 P20 and GSDMD-N in primary microglia. **J-O** Western blot was used to estimate the protein levels of Erbin, Iba-1, NLRP3, Caspase-1 P20 and GSDMD-N in BV2 cells. **P-R** mRNA levels of IL-1β, TNF-α, and IL-6 in BV2 cells were tested by qPCR. **S** Cell viability of HT22 cells was examined by CCK8. Data are representative of three independent experiments. **p*<0.05, ***p<*0.01 vs. Sham or Control or *siNC* group; ^#^*p*<0.05, ^##^*p*<0.01 vs. CLP or LPS/nigericin group or *siNC*+LPS/nigericin group.


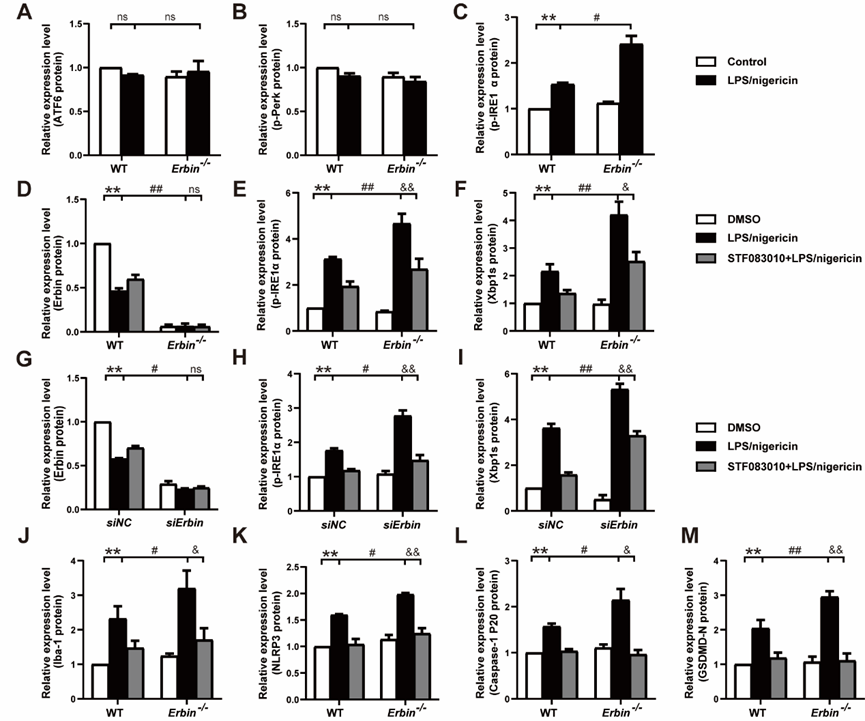


**Fig.S2. Erbin regulates pyroptosis via IRE1α/Xbp1s/Ca^2+^ in vivo and vitro.**

**A-C** Three main ER stress sensors, ATF6, PERK, and IRE1α in primary mocroglia were measured by western blot. **D-F** Added STF083010, Erbin and IRE1α/Xbp1s pathways protein expressions in primary mocroglia were analyzed by western blot. **G-I** Erbin and IRE1α/Xbp1s pathways protein expressions in BV2 cells were analyzed by western blot. **J-M** Western blot was used to estimate the protein levels of Iba-1, NLRP3, Caspase-1 P20 and GSDMD-N in primary microglia. Data are representative of three independent experiments. **p*<0.05, ***p<*0.01 vs. Control or *siNC* group; ^#^*p*<0.05, ^##^*p*<0.01 vs. LPS/nigericin or *siNC*+LPS/nigericin group; ^&^*p*<0.05, *^&&^p<*0.01 vs. *Erbin^-/-^*+LPS/nigericin or *siErbin* +LPS/nigericin group.


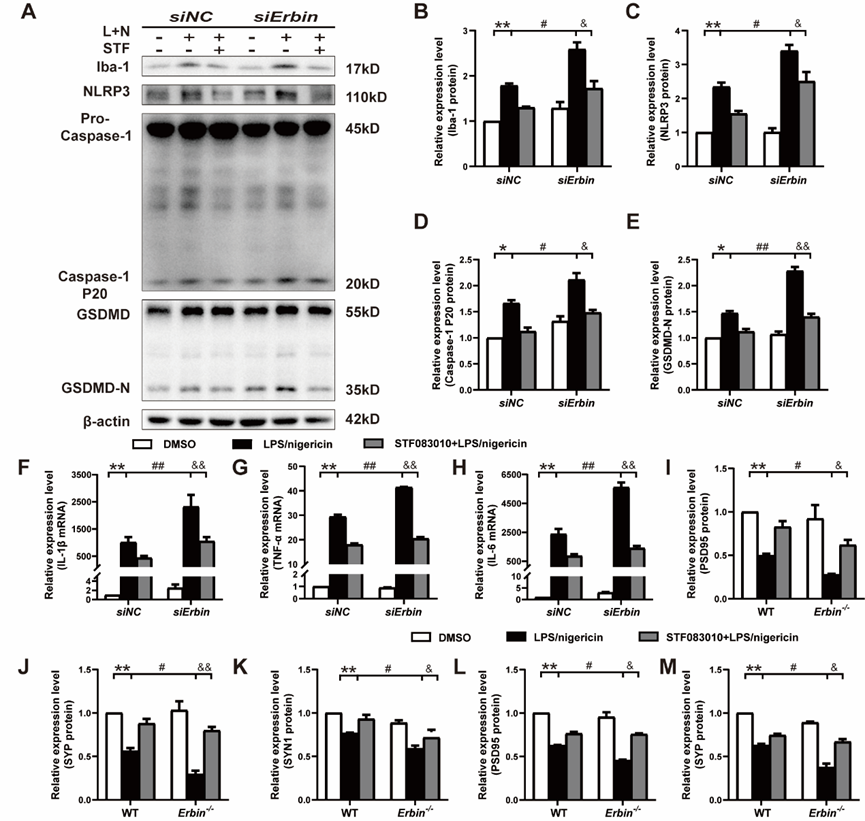


**Fig.S3. Erbin inhibits pyroptosis via IRE1α/Xbp1s/Ca^2+^ in BV2 cells and improves synaptic proteins by inhibiting** **IRE1α/Xbp1s pathway.**

**A-E** Western blot was used to estimate the protein levels of Iba-1, NLRP3, Caspase-1 P20 and GSDMD-N in BV2 cells. **F-H** mRNA levels of IL-1β, TNF-α, and IL-6 in BV2 cells were tested by qPCR. **I-K** Synaptic proteins PSD95, SYP and SYN1 in hippocampus were measured after given STF083010. **L-M** Synaptic proteins PSD95 and SYP in HT22 cells were measured after given STF083010. Data are representative of three independent experiments. **p*<0.05, ***p<0.01* vs. *siNC* or Sham or Control group; ^#^*p*<0.05, ^##^*p*<0.01 vs. *siNC*+LPS/nigericin or CLP or LPS/nigericin group; &p<0.05, &&p<0.01 vs. *siErbin*+LPS/nigericin or STF083010+CLP or *Erbin^-/-^*+LPS/nigericin group.


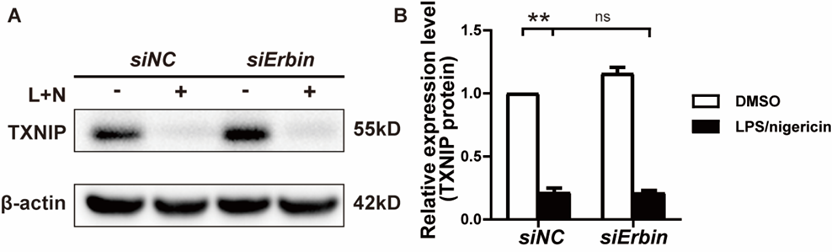


**Fig.S4. LPS****/nigericin stimulation resulted in decreased TXNIP protein levels regardless of *Erbin* knockdown in BV2 cells.**

TXNIP has been demonstrated to bind NLRP3 during irremediable ER stress leading to increased inflammasome activation and cell death during the process of kinds of diseases in previous literature^1-3^. However, our study showed that LPS/nigericin treatment resulted in decreased TXNIP expression whether *Erbin* was knocked down or not in BV2 cells. **A-B** Western blot was used to estimate the protein levels of TXNIP in BV2 cells. ***p*<0.01 vs. *siNC* group.

1. Chen D, Dixon BJ, Doycheva DM, et al. IRE1alpha inhibition decreased TXNIP/NLRP3 inflammasome activation through miR-17-5p after neonatal hypoxic-ischemic brain injury in rats. J Neuroinflammation. 2018;15(1):32

2. Xu W, Li T, Gao L, et al. Apelin-13/APJ system attenuates early brain injury via suppression of endoplasmic reticulum stress-associated TXNIP/NLRP3 inflammasome activation and oxidative stress in a AMPK-dependent manner after subarachnoid hemorrhage in rats. *J Neuroinflammation.* 2019;16(1):247.

3. Szpigel A, Hainault I, Carlier A, et al. Lipid environment induces ER stress, TXNIP expression and inflammation in immune cells of individuals with type 2 diabetes. *Diabetologia.* 2018;61(2):399-412.
